# Sirtuin 5‐Mediated Desuccinylation of ALDH2 Alleviates Mitochondrial Oxidative Stress Following Acetaminophen‐Induced Acute Liver Injury

**DOI:** 10.1002/advs.202402710

**Published:** 2024-08-19

**Authors:** Qiwen Yu, Jiakai Zhang, Jiye Li, Yaodong Song, Jie Pan, Chaopeng Mei, Mengwei Cui, Qianqian He, Haifeng Wang, Huihui Li, Bo Cheng, Yan Zhang, Wenzhi Guo, Changju Zhu, Sanyang Chen

**Affiliations:** ^1^ Department of Emergency Medicine The First Affiliated Hospital of Zhengzhou University Zhengzhou Henan 450052 China; ^2^ Henan Medical Key Laboratory of Emergency and Trauma Research Zhengzhou Henan 450052 China; ^3^ Henan Emergency and Trauma Medicine Engineering Research Center Zhengzhou Henan 450052 China; ^4^ Department of Hepatobiliary and Pancreatic Surgery The First Affiliated Hospital of Zhengzhou University Zhengzhou Henan 450052 China; ^5^ Henan Key Laboratory for Digestive Organ Transplantation The First Affiliated Hospital of Zhengzhou University Zhengzhou Henan 450052 China

**Keywords:** acute liver injury, ALDH2, mitochondrial oxidative stress, SIRT5, succinylation

## Abstract

Acetaminophen (APAP) overdose is a major cause of drug‐induced liver injury. Sirtuins 5 (SIRT5) has been implicated in the development of various liver diseases. However, its involvement in APAP‐induced acute liver injury (AILI) remains unclear. The present study aimed to explore the role of SIRT5 in AILI. SIRT5 expression is dramatically downregulated by APAP administration in mouse livers and AML12 hepatocytes. SIRT5 deficiency not only exacerbates liver injury and the inflammatory response, but also worsens mitochondrial oxidative stress. Conversely, the opposite pathological and biochemical changes are observed in mice with SIRT5 overexpression. Mechanistically, quantitative succinylome analysis and site mutation experiments revealed that SIRT5 desuccinylated aldehyde dehydrogenase 2 (ALDH2) at lysine 385 and maintained the enzymatic activity of ALDH2, resulting in the suppression of inflammation and mitochondrial oxidative stress. Furthermore, succinylation of ALDH2 at lysine 385 abolished its protective effect against AILI, and the protective effect of SIRT5 against AILI is dependent on the desuccinylation of ALDH2 at K385. Finally, virtual screening of natural compounds revealed that Puerarin promoted SIRT5 desuccinylase activity and further attenuated AILI. Collectively, the present study showed that the SIRT5‐ALDH2 axis plays a critical role in AILI progression and might be a strategy for therapeutic intervention.

## Graphical abstract

This study demonstrates the involvement of SIRT5 in APAP‐induced acute liver injury (AILI) by modulating ALDH2‐associated mitochondrial oxidative stress. SIRT5 exhibits decreased expression levels in mouse liver and AML12 hepatocyte models of AILI. Mechanistically, SIRT5 desuccinylates aldehyde dehydrogenase 2 (ALDH2) at lysine 385 and preserves ALDH2 enzymatic activity, moreover, puerarin promoted SIRT5 desuccinylase activity and further attenuated AILI, highlighting the SIRT5‐ALDH2 axis as a promising therapeutic target for AILI intervention.

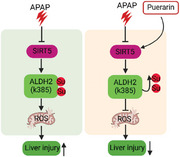

## 1 Introduction

Acetaminophen (APAP), which is a commonly used antipyretic and analgesic drug worldwide, is safe and effective at therapeutic doses.^[^ ^1^ ^]^ However, in recent years, there has been an increase in liver toxicity and injury associated with overdose or inappropriate use of APAP, making it a leading cause of drug‐induced liver injury and acute liver failure.^[^ ^2^, ^3^, ^4^ ^]^ Therefore, it is crucial to explore the mechanisms underlying AILI and to identify new therapeutic methods for this condition.

At therapeutic doses, ≈90% of APAP is transformed into non‐toxic metabolites by glucuronide and sulphation.^[^ ^5^ ^]^ In addition, 5–10% of the substance can be metabolized by cytochrome P450 in the liver into the toxic intermediate N‐acetyl‐p‐benzoquinone imide (NAPQI), which is subsequently converted into a non‐toxic metabolite by glutathione (GSH).^[^ ^6^, ^7^ ^]^ However, an overdose of APAP rapidly reduces GSH levels and causes high levels of NAPQI to bind to mitochondrial membrane proteins, thereby inhibiting mitochondrial respiration and energy metabolism and interfering with the intramitochondrial antioxidant system, leading to changes in mitochondrial membrane permeability and a decrease in membrane potential, resulting in mitochondrial dysfunction.^[^ ^8^, ^9^, ^10^ ^]^ Mitochondria are major sources of ROS and targets of oxidative stress, and ROS overproduction leads to mitochondrial dysfunction, which in turn triggers various pathologies.^[^ ^11^ ^]^ Therefore, maintaining or improving mitochondrial function may be a crucial approach for treating AILI.

Succinylation is a recently discovered posttranscriptional modification of proteins that is involved in regulating many biological processes in cells, such as mitochondrial metabolism, energy production, the oxidative stress response, the immune response, and drug metabolism.^[^ ^12^, ^13^, ^14^, ^15^ ^]^ Succinylation has been closely associated with liver diseases. In liver cancer tissues, succinylation levels are higher than those in adjacent tissues, and higher succinylation is associated with a poorer prognosis.^[^ ^16^ ^]^ Additionally, succinylation levels are significantly increased in nonalcoholic fatty liver disease, and inhibiting the succinylation of related proteins can alleviate liver I/R injury.^[^ ^17^, ^18^ ^]^

The sirtuin family is a class of NAD+‐dependent protein deacetylases that consists of seven members (sirtuin 1 (SIRT1) to SIRT7), which perform important regulatory functions in cells and are involved in a variety of biological processes and disease pathogenesis.^[^ ^19^, ^20^ ^]^ SIRT5 is a member of the sirtuin family that is localized mainly in mitochondria, it has deacetylase, desuccinylase, desglycosylase and desglutarylase activities.^[^ ^21^, ^22^ ^]^ The desuccinylase activity of SIRT5 has significant implications for the structure and function of various proteins, playing crucial roles in numerous diseases and influencing their onset and progression.^[^ ^23^, ^24^ ^]^ Notably, SIRT5 plays an important role in liver diseases. SIRT5 depletion in the liver of mice can accelerate liver I/R injury.^[^ ^17^ ^]^ In contrast, liver overexpression or pharmacological activation of SIRT5 has been shown to exert an obvious hepatoprotective effect against liver I/R injury and steatosis.^[^ ^25^, ^26^ ^]^ However, to our knowledge, no previous studies have explored the role of SIRT5 in AILI.

In the present study, we revealed that SIRT5 was significantly downregulated in AILI and that SIRT5 depletion exacerbated mitochondrial oxidative stress in vivo and in vitro. Mechanistically, SIRT5 desuccinylated aldehyde dehydrogenase 2 (ALDH2) at lysine 385 and maintained the enzymatic activity of ALDH2. Moreover, activating the desuccinylase activity of SIRT5 with puerarin attenuated AILI. Thus, the SIRT5‐ALDH2 axis plays a critical role in AILI and may be an avenue for therapeutic intervention.

## 2 Results

### 2.1 Hepatocyte SIRT5 Expression is Dramatically Downregulated in AILI

**Figure 1:**
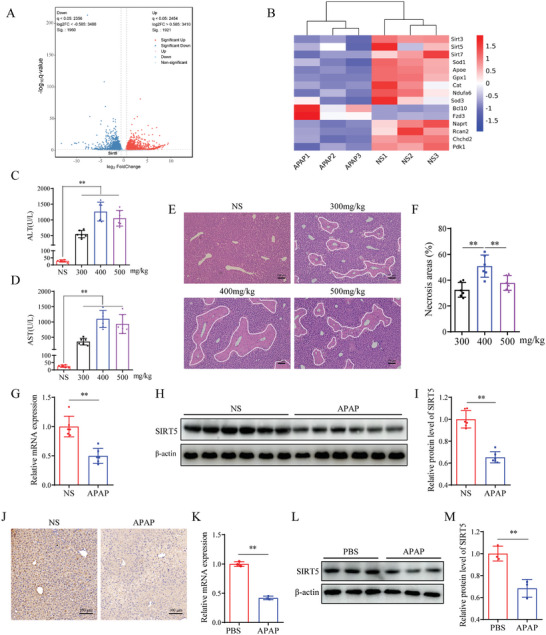
Hepatocyte SIRT5 expression is dramatically downregulated in AILI. A) Volcano plot of differentially mRNA expression between the NS and APAP treated mice. B) Heat map of differentially mRNA expression between the NS and APAP treated mice. C, D) Serum ALT and AST levels of different doses APAP treated mice (n = 6). E, F) H&E staining and necrotic area statistics of liver tissues (n = 6). Scale bar, 100 µm. G) *Sirt5* mRNA expression in liver tissues treated with NS and APAP (n = 6). H, I) SIRT5 protein expression and statistical analysis in liver tissues treated with NS and APAP (n = 6). J) SIRT5 immunohistochemical staining of liver tissues treated with NS and APAP (n = 6). Scale bar, 100 µm. K) *Sirt5* mRNA expression in AML12 hepatocytes after 24h of APAP treatment (n = 3). L, M) SIRT5 protein expression and statistical analysis in AML12 hepatocytes after 24h of APAP treatment (n = 6). All data are presented as the mean ± SD. Levels of statistical significance are indicated as *P < 0.05, **P < 0.01; ns, not significant. One‐way ANOVA with Tukey test analysis and a two‐tailed Student t test were used for statistical analysis.

RNA sequencing data of control and APAP‐treated mouse liver tissues showed that SIRT5 expression in liver tissues was significantly downregulated after APAP administration (**Figure** **1**A,B). To verify whether SIRT5 is involved in AILI, C57BL/6 mice were treated with different doses of APAP for 24 h. Compared with those in the control group, serum ALT and AST levels were increased to varying degrees in all APAP‐treated mice and peaked in the 400 mg kg^−1^ APAP group (Figure 1C,D). H&E staining also showed that necrosis was most severe in the 400 mg kg^−1^ APAP group (Figure 1E,F). Therefore, we selected 400 mg/kg APAP for subsequent experiments. Consistent with the RNA sequencing data, a marked decrease in SIRT5 was observed in the APAP group compared with the control group (Figure 1G). Western blotting and immunohistochemistry revealed the downregulation of SIRT5 in the liver after APAP administration (Figure 1H‐J). Additionally, AML12 cells were treated with different concentrations of APAP for 24 h, cellular activity was determined using the CCK‐8 assay, and 20 mM APAP was selected for cellular experiments (Figure S1, Supporting Information), then the expression of SIRT5 was detected. We observed that the mRNA and protein expression of SIRT5 in AML12 hepatocytes was downreguled by APAP (Figure 1K‐M). In summary, these findings indicate that SIRT5 is a key mediator of the development of AILI.

### 2.2 SIRT5 Ameliorates APAP‐Induced Hepatotoxicity

**Figure 2:**
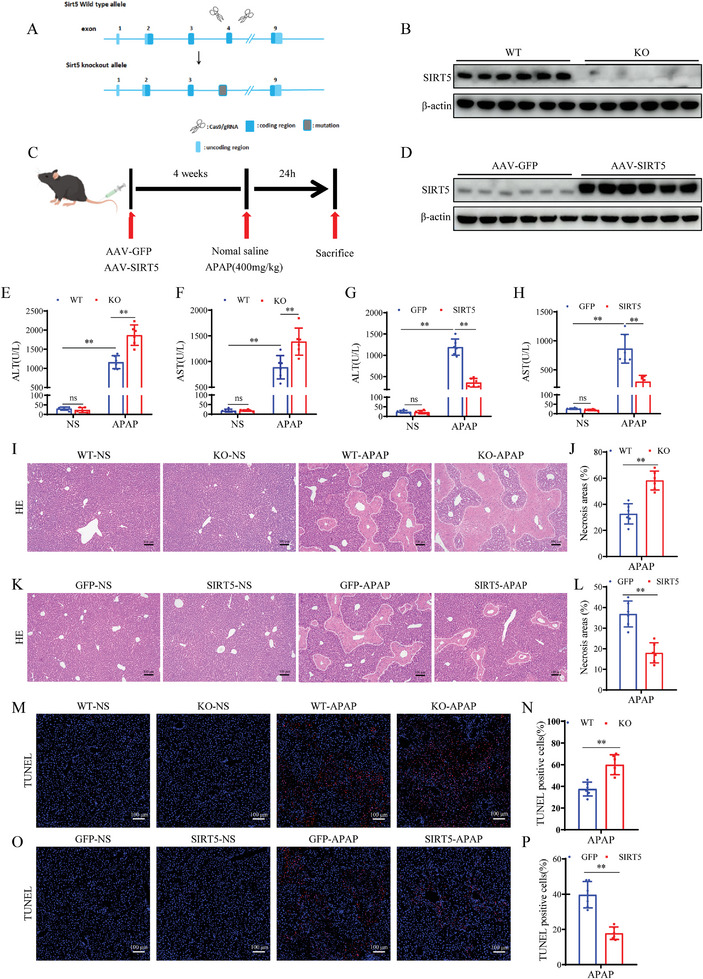
SIRT5 ameliorates APAP‐induced liver hepatotoxicity. A, B) Diagram of the SIRT5‐KO strategy and SIRT5 protein expression in livers tissues of WT and SIRT5‐KO mice (n = 6). C, D) Schematic representation of adeno‐associated virus intervention in mice and SIRT5 protein expression in livers tissues of AAV‐GFP and AAV‐SIRT5 mice (n = 6). E, F) Serum ALT/AST levels in WT and SIRT5‐KO mice treated with APAP for 24 h (n = 6). G, H) Serum ALT/AST levels in AAV‐GFP and AAV‐SIRT5 mice treated with APAP for 24 h (n = 6). I, J) H&E staining and necrotic area statistics of liver tissues in WT and KO mice treated with APAP for 24 h (n = 6). Scale bar, 100 µm. K, L) H&E staining and necrotic area statistics of liver tissues in AAV‐GFP and AAV‐SIRT5 mice treated with APAP for 24 h (n = 6). Scale bar, 100 µm. M, N) TUNEL staining and statistical analysis of liver tissue in WT and KO mice treated with APAP for 24 h (n = 6). Scale bar, 100 µm. O, P) TUNEL staining and statistical analysis of liver tissue in AAV‐GFP and AAV‐SIRT5 mice treated with APAP for 24 h (n = 6). Scale bar, 100 µm. All data are presented as the mean ± SD. Levels of statistical significance are indicated as *P < 0.05, **P < 0.01; ns, not significant. One‐way ANOVA with Tukey test analysis and a two‐tailed Student t test were used for statistical analysis.

Based the significant downregulation of SIRT5 expression in AILI, we constructed SIRT5‐KO mice and mice with AAV‐mediated liver‐specific SIRT5 overexpression to further investigate the role of SIRT5 in AILI (**Figure** **2**A,C). The western blot results confirmed KO and overexpression of SIRT5 in the liver (Figure 2B,D). After APAP administration, serum ALT and AST levels were significantly increased in WT mice; and these increases were more pronounced in SIRT5‐KO mice (Figure 2E,F). H&E staining of liver sections revealed that APAP‐induced necrosis was significantly aggravated in the SIRT5‐KO mice (Figure 2I,J). Intriguingly, AAV‐mediated liver‐specific SIRT5 overexpression significantly improved the liver damage in response to APAP (Figure 2G,H,K‐L). TUNEL staining analysis demonstrated a higher incidence of hepatocellular death in SIRT5‐KO mice following APAP administration compared to WT mice (Figure 2M,N). Conversely, SIRT5 overexpression mice exhibited a significant decrease in hepatocellular death (Figure 2O,P). Furthermore, in vitro cellular experiments also demonstrated the protective role of SIRT5 against APAP‐induced toxicity (Figure S2, Supporting Information). Taken together, these results demonstrate that SIRT5 ameliorates APAP‐induced hepatotoxicity.

### 2.3 SIRT5 Inhibits APAP‐Induced Liver Inflammation

**Figure 3:**
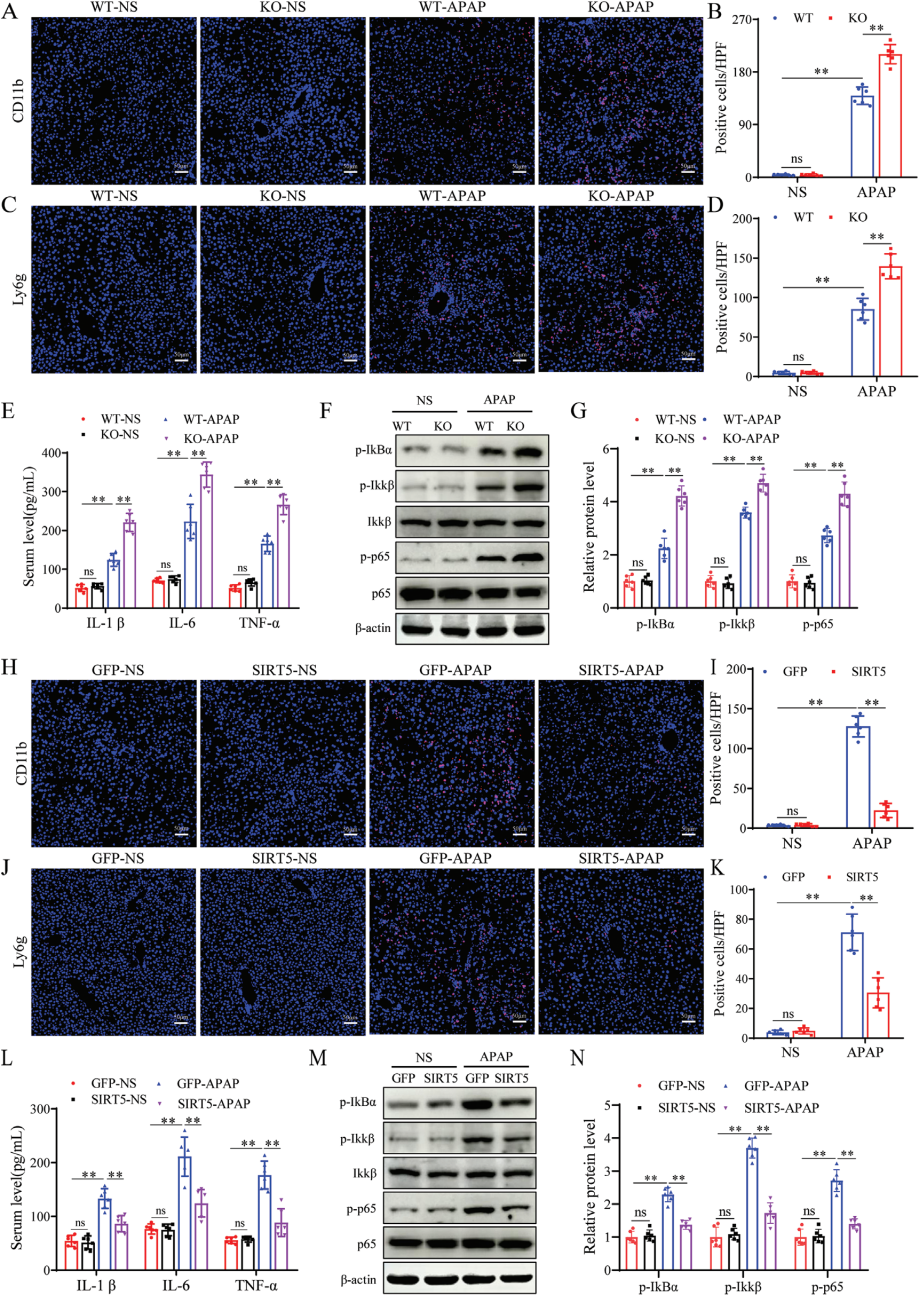
SIRT5 inhibits APAP‐induced liver inflammation. A, B) Immunofluorescence staining of CD11b‐positive inflammatory cells (red) and statistics of liver tissue in WT and KO mice treated with APAP for 24 h (n = 6). Scale bar, 50 µm. C, D) Immunofluorescence staining of Ly6g‐positive inflammatory cells (red) and statistics of liver tissue in WT and KO mice treated with APAP for 24 h (n = 6). Scale bar, 50 µm. E) Serum levels of inflammatory cytokines IL‐1β, IL‐6, and TNF‐α in WT and SIRT5‐KO mice treated with APAP for 24 h (n = 6). F, G) NF‐κB signaling protein detection and statistical analysis in liver tissues of WT and KO mice treated with APAP for 24 h (n = 6). H, I) Immunofluorescence staining of CD11b‐positive inflammatory cells (red) and statistics of liver tissue in AAV‐GFP and AAV‐SIRT5 mice treated with APAP for 24 h (n = 6). Scale bar, 50 µm. J, K) Immunofluorescence staining of Ly6g‐positive inflammatory cells (red) and statistics of liver tissue in AAV‐GFP and AAV‐SIRT5 mice treated with APAP for 24 h (n = 6). Scale bar, 50 µm. L) Serum levels of inflammatory cytokines IL‐1β, IL‐6, and TNF‐α in AAV‐GFP and AAV‐SIRT5 mice treated with APAP for 24 h (n = 6). M, N) NF‐κB signaling protein detection and statistical analysis in liver tissues of WT and KO mice treated with APAP for 24 h (n = 3). All data are presented as the mean ± SD. Levels of statistical significance are indicated as *P < 0.05, **P < 0.01; ns, not significant. One‐way ANOVA with Tukey test analysis and a two‐tailed Student *t* test were used for statistical analysis.

Several studies have reported that the hepatotoxicity induced by APAP is closely related to inflammation. In this study, Immunofluorescence staining revealed that the number of CD11b‐ and Ly6g‐ positive inflammatory cells were significantly increased in SIRT5‐KO mice subjected to APAP administration compared to WT mice (**Figure** **3**A‐D). In addition, the levels of inflammatory cytokines (IL‐1β, IL‐6, and TNF‐α) were dramatically increased in the livers of SIRT5‐KO mice compared to those of WT mice (Figure 3E). Moreover, the activation of NF‐κB signaling was increased in SIRT5‐KO mice compared to WT mice with AILI (Figure 3F,G). In contrast to SIRT5 KO, liver‐specific SIRT5 overexpression mice exhibited reduced CD11b and Ly6g inflammatory cell infiltration (Figure 3H‐K), lower serum inflammatory cytokine (Figure 3L), and reduced NF‐κB activation compared with that in the AAV‐GFP group mice (Figure 3M,N). Taken together, these results demonstrate that SIRT5 inhibits APAP‐induced liver inflammation in AILI.

### 2.4 SIRT5 Inhibits APAP‐Induced Mitochondrial Oxidative Stress in AILI

**Figure 4:**
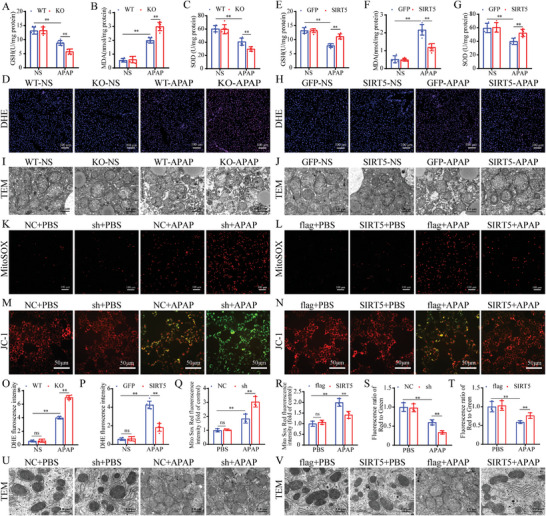
SIRT5 represses APAP‐induced mitochondrial oxidative stress in AILI. A‐C) Liver GSH, MDA, and SOD level in WT and SIRT5‐KO mice treated with APAP for 24 h (n = 6). D, O) DHE staining of liver tissues in WT and SIRT5‐KO mice treated with APAP for 24 h (n = 6). Scale bar, 100 µm. E, G) Liver GSH, MDA, and SOD level in AAV‐GFP and AAV‐SIRT5 mice treated with APAP for 24 h (n = 6). H, P) DHE staining of liver tissues in AAV‐GFP and AAV‐SIRT5 mice treated with APAP for 24 h (n = 6). Scale bar, 100 µm. I) Mitochondrial structure of WT and SIRT5‐KO mice were observed by TEM after 24 h of APAP treatment. Scale bar, 1 µm. J) Mitochondrial structure of AAV‐GFP and AAV‐SIRT5 mice were observed by TEM after 24 h of APAP treatment. Scale bar, 1 µm. K, Q) MitoSOX staining of SIRT5 control and knockdown AML12 hepatocytes after 24 h treatment with APAP (n = 3). Scale bar, 100 µm. L, R) MitoSOX staining of flag and SIRT5 overexpress AML12 hepatocytes after 24 h treatment with APAP (n = 3). Scale bar, 100 µm. M, S) JC‐1 mitochondrial staining of SIRT5 control and knockdown AML12 hepatocytes after 24 h treatment with APAP (n = 3). Scale bar, 50 µm. N, T) JC‐1 mitochondrial staining of SIRT5 flag and SIRT5 overexpress AML12 hepatocytes after 24 h treatment with APAP (n = 3). Scale bar, 50 µm. U) Mitochondrial structure of SIRT5 control and knockdown AML12 hepatocytes were observed by TEM after 24 h of APAP treatment. Scale bar, 1 µm. V) Mitochondrial structure of flag and SIRT5 overexpress AML12 hepatocytes were observed by TEM after 24 h of APAP treatment. Scale bar, 1 µm. All data are presented as the mean ± SD. Levels of statistical significance are indicated as *P < 0.05, **P < 0.01; ns, not significant. One‐way ANOVA with Tukey test analysis and a two‐tailed Student t test were used for statistical analysis.

During AILI, excess toxic response metabolite NAPQI produced by cytochrome P450 enzymes depletes GSH and covalently binds to mitochondrial proteins to form APAP adducts, which leads to mitochondrial dysfunction, the generation of ROS, and the release of mitochondrial cell death factors, ultimately resulting in hepatocyte death. We investigated the effects of SIRT5 KO or overexpression on the expression of the key cytochrome P450 enzymes of APAP metabolism, Cyp2e1, Cyp1a2, and Cyp1a2, and found that SIRT5 KO or overexpression had no significant effect on the expression of these enzymes (Figure S3, Supporting Information). We then explored the effect of SIRT5 on APAP‐induced mitochondrial oxidative stress. To evaluate the effect of SIRT5 on liver mitochondrial oxidative stress after APAP administration, the levels of GSH, MDA, and GSH were measured. APAP administration resulted in significant increases in MDA levels and decreases in SOD and GSH levels, and these changes were significantly deteriorated in the livers of SIRT5‐KO mice (**Figure** **4**A‐C). To further confirm that SIRT5 affects APAP‐induced liver ROS production, liver sections were stained with DHE. The results showed that APAP administration significantly increased DHE fluorescence intensity in the liver sections of WT mice, but the liver sections of SIRT5‐KO mice exhibited higher DHE intensity than those of WT mice (Figure 4D,O). Moreover, TME showed that mitochondrial cristae disappeared and that the mitochondrial membrane was ruptured more obviously after SIRT5 KO (Figure 4I). In contrast, SIRT5 overexpression significantly suppressed mitochondrial oxidative stress, improved mitochondrial cristae loss and membrane rupture caused by APAP (Figure 4E‐H, J,P). In addition, the detection of GSH, SOD, and MDA at 6 and 12 h after APAP administration also indicated the inhibitory effect of SIRT5 on APAP‐induced mitochondrial oxidative stress (Figure S4, Supporting Information). Further in vitro results indicated that SIRT5 knockdown in AML12 hepatocytes markedly increased APAP‐induced mitochondrial ROS production and the loss of mitochondrial membrane potential (Figure 4K,Q, M,S). Additionally, SIRT5 knockdown markedly increased APAP‐induced mitochondrial swelling and cristae breakage in AML12 hepatocytes (Figure 4U). Similarly, SIRT5‐overexpressing AML12 hepatocytes exhibited the opposite changes after APAP administration (Figure 4L, R, N, T, V). Taken together, these in vivo and in vitro results reveal that SIRT5 inhibits mitochondrial oxidative stress during AILI.

### 2.5 SIRT5 Deficiency Induces a Global Increase in Protein Succinylation in AILI

**Figure 5:**
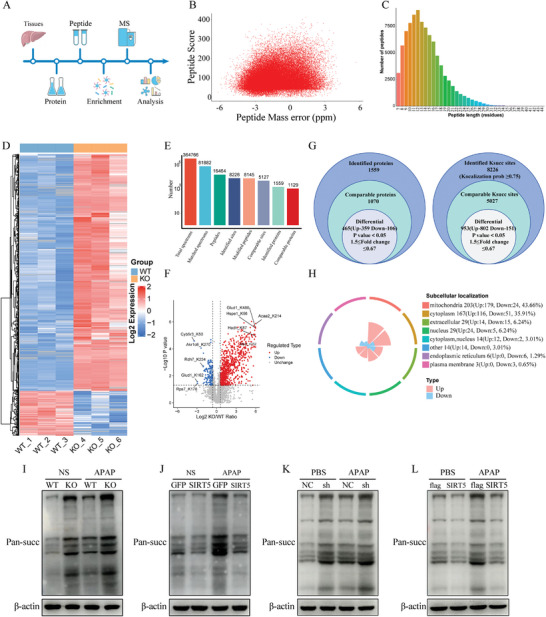
SIRT5 deficiency induces a global increase of protein succinylation in AILI. A) Experimental workflow for the proteomic quantification of Ksuc protein and site. B) The distribution of peptide scores. C) The distribution of tryptic peptide lengths. D) Heat map of differentially succinylated modifification sites between the WT and KO groups. E) Basic statistical figure of MS results. F) Volcano plot of differentially succinylated modifification sites between the WT and KO mice. G) Venn diagram showing the total numbers of Ksuc sites identifified in WT and KO mice liver tissues. H) Subcellular localization of identified succinylated proteins. I) Total succinylation level of protein in liver tissue between the WT and KO groups (n = 6). J) Total succinylation level of protein in liver tissue between the AAV‐GFP and AAV‐SIRT5 groups (n = 6). K) Total succinylation level of protein in SIRT5 control and knockdown AML12 hepatocytes (n = 3). L) Total succinylation level of protein in SIRT5 flag and SIRT5 overexpress AML12 hepatocytes (n = 3).

Given the well‐defined function of SIRT5 in desuccinylation, LC–MS/MS analysis was used to systematically analyze succinylation in the livers of APAP‐treated WT and SIRT5‐KO mice (**Figure** **5**A). The average mass error for all identified peptides was nearly zero, and the errors were consistently less than 5 ppm, indicating that the MS data met the required level of mass accuracy (Figure 5B). Moreover, the majority of peptide lengths fell within the expected range of 8 to 20, corresponding to tryptic peptides and confirming that our sample preparation adhered to the standards for proteomics analysis (Figure 5C). LC–MS/MS analysis identified a total of 8,226 lysine succinylation sites in 1559 proteins, and 5127 sites in 1129 proteins contained quantitative information (Figure 5D,E). Quantitative data with a ratio >1.5 or <0.667 and a p value <0.05 were considered to indicate differential succinylation (Figure 5F). A total of 953 sites in 465 proteins that exhibited differential succinylation between APAP‐treated WT and KO mouse liver tissues (Figure 5G). Among these sites, 802 sites in 359 proteins showed increased succinylation levels, while 151 sites in 106 proteins showed decreased succinylation levels (Figure 5G). Cellular component analysis showed many succinylated proteins that were localized to mitochondria and cytoplasm (Figure 5H).

Global succinylome analyses showed that SIRT5 deficiency altered succinylation levels in AILI, which was further verified in vivo and in vitro. The results revealed that the total succinylation level of proteins in liver tissue and AML12 hepatocytes was increased after APAP administration and further increased in the liver tissue of SIRT5‐KO mice (Figure 5I) and in AML12 hepatocytes with SIRT5 knockdown (Figure 5K). However, overexpression of SIRT5 inhibited APAP‐induced total succinylation of protein (Figure 5J) and AML12 hepatocytes (Figure 5L). These results indicate that SIRT5 deficiency induces a global increase in protein succinylation in AILI.

### 2.6 SIRT5 Desuccinylates ALDH2 at the K385 Residue

**Figure 6:**
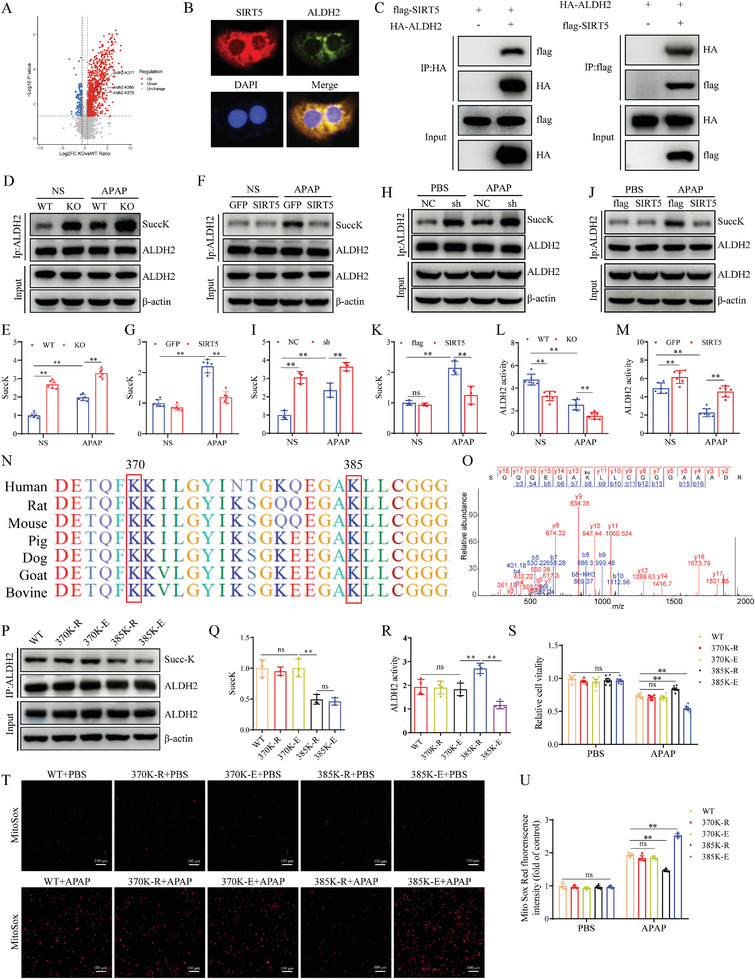
SIRT5 desuccinylates ALDH2 at the K385 residues. A) Volcano plot of ALDH2 succinylated modifification sites between the WT and KO groups. B) Representative fluorescence images showing colocalization between SIRT5 (red) and ALDH2 (green) in AML12 hepatocytes. C) Flag‐tagged SIRT5 and HA‐tagged ALDH2 plasmids were cotransfected into HEK‐293T cells. Anti‐HA antibody (left panel) and anti‐Flag antibody (right panel) were used for immunoprecipitation. D‐E) Expression and statistical analysis of succinylated ALDH2 protein of liver tissues in WT and KO mice treated with APAP for 24 h (n = 6). F‐G) Expression and statistical analysis of succinylated ALDH2 protein of liver tissues in AAV‐GFP and AAV‐SIRT5 mice treated with APAP for 24 h (n = 6). H‐I) Expression and statistical analysis of succinylated ALDH2 protein in SIRT5 control and knockdown AML12 hepatocytes after 24 h treatment with APAP (n = 3). J‐K) Expression and statistical analysis of succinylated ALDH2 protein in flag and SIRT5 overexpress AML12 hepatocytes after 24 h treatment with APAP (n = 3). L‐M) ALDH2 activity after 24 h treatment with APAP in each group (n = 6). N) The K370 and K385 site are highly conserved in different species, ranging from mice to humans. O) LC‒MS/ MS spectrum of the succinylated ALDH2 K385 site. P‐Q) Expression of and statistical analysis of succinylated ALDH2 protein in AML12 hepatocytes after transfection with different plasmids (n = 3). R) ALDH2 activity in AML12 hepatocytes transfected with different plasmids (n = 4). S) Cell activity of AML12 hepatocytes transfected with different plasmids (n = 6). T, U) MitoSOX staining in AML12 hepatocytes transfected with different plasmids (n = 3). Scale bar, 100 µm. All data are presented as the mean ± SD. Levels of statistical significance are indicated as *P < 0.05, **P < 0.01; ns, not significant. One‐way ANOVA with Tukey test analysis and a two‐tailed Student *t* test were used for statistical analysis.

Among the protein succinylation levels caused by SIRT5 deficiency, the succinylation of ALDH2, a critical enzyme involved in mitochondrial oxidative stress, was significantly upregulated (**Figure** **6**A). To explore the exact molecular mechanism by which SIRT5 regulates ALDH2 succinylation, we examined the localization of SIRT5 and ALDH2, and found that SIRT5 colocalized with ALDH2 (Figure 6B). Coimmunoprecipitation experiments showed that SIRT5 interacted with ALDH2 (Figure 6C). Furthermore, ablation of SIRT5 significantly upregulated the succinylation level of ALDH2 in vivo and in vitro but had no effect on the total protein concentration of ALDH2 (Figure 6D,E,H,I). Conversely, SIRT5 overexpression significantly decreased the succinylation level of ALDH2 (Figure 6F,G, J,K). The enzymatic activity of ALDH2 is crucial for its effectiveness, and we examined whether SIRT5 affected the activity of ALDH2. Interestingly, SIRT5 deficiency inhibited the enzymatic activity of ALDH2, and SIRT5 overexpression increased the activity of ALDH2 (Figure 6L,M).

Next, LC‒MS/MS showed that the succinylation of three sites in ALDH2 (K370, K377, K385) was significantly increased (Figure 6A, O). Among these sites, Lys370 (K370) and Lys385 (K385) are highly conserved in different species from human to bovine (Figure 6M). We subsequently generated K370 and K385 mutant plasmids (370K‐R, 370K‐E, 385K‐R, and 385K‐E), lysine (K) mutated to glutamic acid (E) mimicking succinylation, and K mutated to arginine (R) mimicking desuccinylation, and transfected them into AML12 hepatocytes. Of these mutants, the 385K‐R and 385K‐E mutants showed obviously lower levels of succinylation, indicating that K385 is the key succinylation site on ALDH2 (Figure 6P,Q). Since K385 is the key succinylation site on ALDH2, we further evaluated whether K385 succinylation modulates ALDH2 enzymatic activity. AML12 hepatocytes, transfected with 370K‐R and 370K‐E plasmids had no effect on ALDH2 activity, while hepatocytes transfected with 385K‐R significantly increase the activity of ALDH2 and hepatocytes transfected with 385K‐E suppressed ALDH2 activity (Figure 6R). These data indicated that succinylation at K385 but not K370 affected the enzymatic activity of ALDH2.

Moreover, the CCK‐8 and MitoSOX staining results indicated that there was no change in cell viability or mitochondrial ROS levels between hepatocytes transfected with the ALDH2 WT, ALDH2‐370K‐R or ALDH2‐370K‐E plasmids after APAP administration. However, hepatocytes transfected with ALDH2‐385K‐R plasmids had markedly increased cell viability and reduced mitochondrial ROS levels compared to hepatocytes transfected with ALDH2 WT plasmids; hepatocytes transfected with ALDH2‐385K‐E plasmids had decreased viability and increased mitochondrial ROS generation (Figure 6S‐U). These results evidenced that SIRT5 alleviated AILI mainly through the desuccinylation of ALDH2 at the K385 residue.

### 2.7 Desuccinylation of ALDH2 at the K385 Residue Protects Mice from AILI

**Figure 7:**
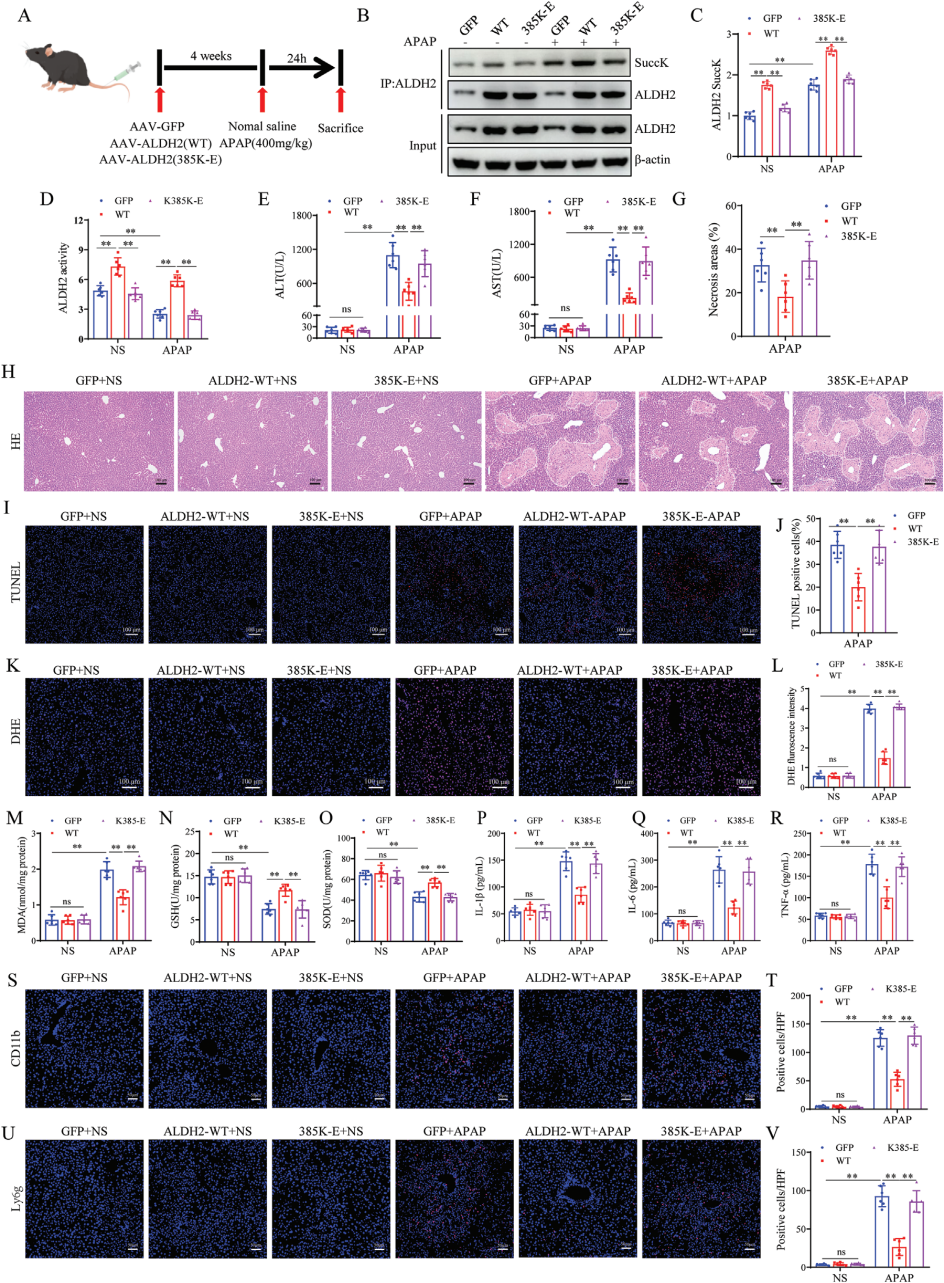
Desuccinylation of ALDH2 at K385 residue protects mice from AILI. A) Schematic representation of Adeno‐associated virus intervention in mice. B‐C) The expression and statistical analysis of succinylated ALDH2 protein in liver tissue of different groups indicated after 24 h of APAP (n = 6). D) ALDH2 activity after 24 h treatment with APAP in each group. E‐F) Serum ALT/AST levels of the mice described in (A) (n = 6). G‐H) H&E staining and necrotic area statistics of liver tissues in mice described in (A) (n = 6). Scale bar, 100 µm. I‐J) TUNEL staining and statistical analysis of liver tissues in mice described in (A) (n = 6). Scale bar, 100 µm. K‐L) DHE staining and necrotic area statistics of liver tissues in mice described in (A) (n = 6). Scale bar, 100 µm. M‐O) Liver tissues GSH, MDA, and SOD level of different groups indicated after 24 h of APAP (n = 6). P‐R) Serum levels of inflammatory cytokines TNF‐α, IL‐1β, and IL‐6 in mice described in (A) (n = 6). Scale bar, 50 µm. S‐V) Immunofluorescence staining of CD11b‐ and Ly6g‐positive inflammatory cells (red) and statistics of liver tissue in mice described in (A) (n = 6). Scale bar, 50 µm. All data are presented as the mean ± SD. Levels of statistical significance are indicated as *P < 0.05, **P < 0.01; ns, not significant. One‐way ANOVA with Tukey test analysis and a two‐tailed Student *t* test were used for statistical analysis.

To examine the role of ALDH2‐K385 desuccinylation in AILI, AAV‐GFP, AAV‐ALDH2‐WT, and AAV‐ALDH2‐385K‐E overexpressed transfected mice were generated and then subjected to APAP challenge (**Figure** **7**A). The data indicated that APAP administration increased the succinylation of ALDH2, while the succinylation of ALDH2 was lower in the livers of ALDH2‐385K‐E mice compared to that in ALDH2‐WT mice (Figure 7B,C). The activity assay showed that the activity of ALDH2 decreased after APAP administration, and in contrast to that in ALDH2‐WT mice, the activity of ALDH2 was reduced in ALDH2‐385K‐E mice (Figure 7D). In addition, the transaminase (ALT/AST) levels, liver necrotic areas, and hepatocellular death were increased in ALDH2‐385K‐E mice compared to ALDH2‐WT mice after APAP administration (Figure 7E‐J). Moreover, APAP‐induced mitochondrial oxidative stress and inflammation were aggravated in ALDH2‐385K‐E mice compared to the ALDH2‐WT mice after APAP administration (Figure 7K‐V). Collectively, these datas suggest that desuccinylation of ALDH2 at the K385 residue protects mice from AILI.

### 2.8 Desuccinylation of ALDH2 at K385 Mediates the Protective Effect of SIRT5 against AILI

**Figure 8:**
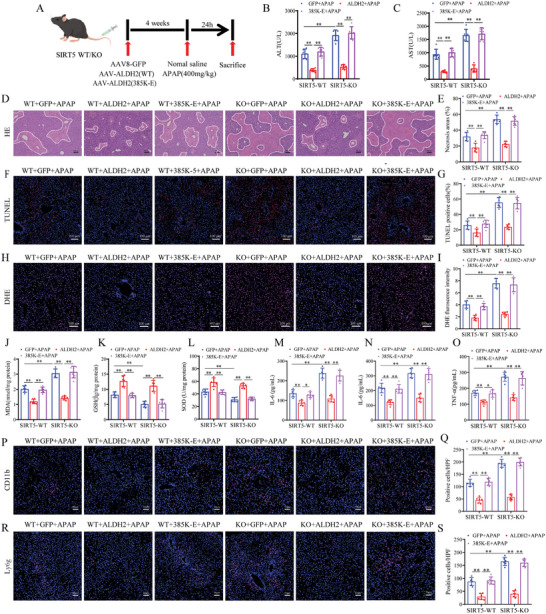
Desuccinylation of ALDH2 at K385 mediates the protective effect of SIRT5 in AILI. A) Schematic representation of Adeno‐associated virus intervention in mice. B‐C) Serum ALT and AST levels of the mice described in (A) (n = 6). D, E) H&E staining and necrotic area statistics of liver tissues in mice described in (A) (n = 6). Scale bar, 100 µm. F, G) TUNEL staining and statistical analysis of liver tissues in mice described in (A) (n = 6). Scale bar, 100 µm. H, I) DHE staining and necrotic area statistics of liver tissues in mice described in (A) (n = 6). Scale bar, 100 µm. J‐JL) Liver tissues GSH, MDA and SOD level of different groups indicated after 24 h of APAP (n = 6). M‐O) Serum levels of inflammatory cytokines TNF‐α, IL‐1β, and IL‐6 in mice described in (A) (n = 6). Scale bar, 50 µm. P‐S) Immunofluorescence staining of CD11‐b and Ly6g‐positive inflammatory cells (red) and statistics of liver tissue in mice described in (A) (n = 6). Scale bar, 50 µm. All data are presented as the mean ± SD. Levels of statistical significance are indicated as *P < 0.05, **P < 0.01; ns, not significant. One‐way ANOVA with Tukey test analysis and a two‐tailed Student *t* test were used for statistical analysis.

To investigate the role of ALDH2 desuccinylation by SIRT5 in AILI in vivo, we overexpressed the various forms of ALDH2 in SIRT5‐KO mice by injecting the relevant AAVs expressing AAV‐GFP, AAV‐ALDH2‐WT, or AAV‐ALDH2‐385K‐E through the tail vein, these mice were subsequently subjected to APAP administration (**Figure** **8**A). SIRT5 deficiency robustly increased serum transaminase (ALT/AST) levels, induced necrosis and hepatocellular death after APAP administration, and overexpression of ALDH2‐WT significantly ameliorated liver damage (Figure 8B‐G). In APAP‐treated WT and SIRT5‐KO mice, neither the AAV‐GFP nor the AAV‐ALDH2‐385K‐E group exhibited obvious improvements in serum transaminase (ALT/AST) levels, liver necrotic areas, or hepatocellular death compared with those in the AAV‐ALDH2‐WT group (Figure 8B‐G). Moreover, in contrast to those in the AAV‐GFP mice, hepatic oxidation and inflammation were markedly decreased in ALDH2‐WT mice, but not in ALDH2‐385KE mice (Figure 8H‐S). Collectively, these data suggest that desuccinylation of ALDH2 at K385 mediates the protective effect of SIRT5 against AILI.

### 2.9 Pharmacological Promotion of SIRT5 Attenuates AILI

**Figure 9:**
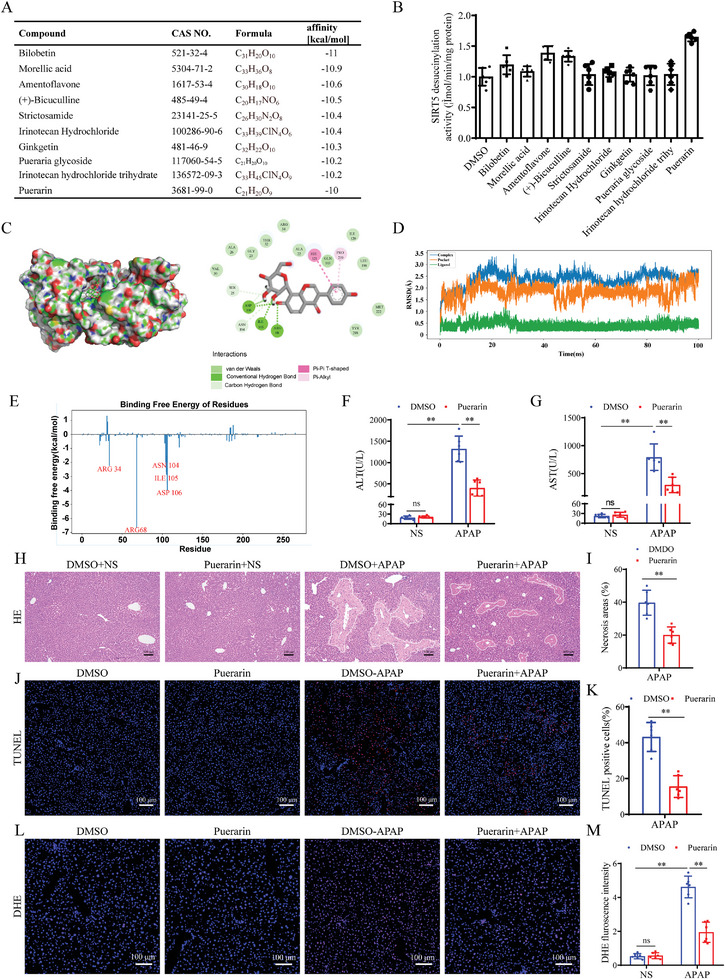
Pharmacological promoting SIRT5 attenuates AILI. A) Docking results of potential compounds and SIRT5. (B) SIRT5 desuccinylase activity in liver tissues of mice after different treatments. C) Putative interactions between SIRT5 and Puerarin. D) The RMSD of the backbone atoms of the SIRT5‐ligand systems for the SIRT5 pocket, Puerarin ligand, and the SIRT5‐Puerarin complex. E) Amino acid energy decomposition of molecular dynamics simulation. F‐G) Serum ALT/AST activities in DMSO and Puerarin treated mice at 24h after APAP treatment (n = 6). H‐I) H&E staining and necrotic area statistics in DMSO and Puerarin treated mice at 24h after APAP treatment (n = 6). Scale bar, 100 µm. J‐K) TUNEL staining and statistic analysis in DMSO and Puerarin treated mice at 24h after APAP treatment (n = 6). Scale bar, 100 µm. (L‐M) DHE staining and fluorescence intensity analysis in DMSO and Puerarin treated mice at 24h after APAP treatment (n = 6). Scale bar, 100 µm. All data are presented as the mean ± SD. Levels of statistical significance are indicated as *P < 0.05, **P < 0.01; ns, not significant. One‐way ANOVA with Tukey test analysis and a two‐tailed Student *t* test were used for statistical analysis.

To explore the therapeutic effects of a SIRT5 activator on AILI, we searched for natural compounds that could bind to SIRT5 through virtual screening. According to the docking results, ten compounds with the lowest affinity energy were selected, and their effects on the activity of SIRT5 desuccinylase were further examined (**Figure** **9**A), puerarin resulted in the most robust increase in SIRT5 desuccinylase activity (the treatment of different drugs can be found in the Supporting Information) (Figure 9B). Molecular docking analysis revealed that SIRT5 could bind to puerarin (Figure 9C). Moreover, molecular dynamics simulation confirmed the binding stability and dynamics of the SIRT5‐puerarin complex at the atomic level (Figure 9D,E). We further verified the effect of puerarin on APAP‐induced liver injury in vivo. Intriguingly, compared with those in the DMSO APAP group, serum AST and ALT levels in the puerarin group were reduced after APAP challenge (Figure 9F,G). H&E staining and and TUNEL staining revealed a reduction in necrosis and hepatocellular death in liver sections from mice treated with puerarin after APAP challenge (Figure 9H‐K). DHE staining and CD11b‐ and Ly6g immunofluorescence staining also revealed that puerarin inhibited APAP‐induced oxidative stress (Figure 9L,M) and inflammation (Figure S5, Supporting Information), while there was no significant effect on the expression of Cyp2e1, Cyp1a2 and Cyp1a2, key cytochrome P450 enzymes for APAP metabolism (Figure S5, Supporting Information). In summary, these findings strongly indicate that pharmacological activation of SIRT5 by puerarin attenuates AILI, suggesting that puerarin is a promising agent for the clinical treatment of AILI.

## 3 Discussion

Although SIRT5 has been implicated in a variety of liver diseases, its specific role and underlying mechanisms in AILI have not been well elucidated. In the present study, we observed a decrease in SIRT5 levels in APAP‐treated mice and AML12 hepatocytes. Gain‐ and loss‐of‐function experiments revealed that SIRT5 played a protective role in AILI by modulating mitochondrial oxidative stress. Mechanistically, SIRT5 inhibited the succinylation of the lysine 385 residue in ALDH2 and increased its enzymatic activity, resulting in the suppression of mitochondrial oxidative stress. Moreover, we found that puerarin promoted SIRT5 desuccinylase activity and further attenuated AILI. Taken together, our findings demonstrate for the first time that targeting the SIRT5‐ALDH2 axis may be a potential therapeutic approach for AILI.

Mitochondrial oxidative stress is the key initiator of APAP‐induced liver injury, and mitochondria are a major source of ROS production in cells.^[^ ^27^, ^28^ ^]^ The interaction between mitochondrial ROS and mitochondrial dysfunction forms a negative feedback loop that accelerates the progression of AILI.^[^ ^8^, ^29^ ^]^ In addition, excessive mitochondrial ROS activate inflammatory signaling pathways to exacerbate AILI.^[^ ^30^, ^31^ ^]^ Inhibition of mitochondrial oxidative stress and the preservation of mitochondrial function are essential for cell survival and improvement of liver function after AILI.^[^ ^32^, ^33^ ^]^ SIRT5 has been implicated in the regulation of mitochondrial oxidative stress. SIRT5 activates the Nrf2/HO‐1 signaling pathway to decrease mitochondrial ROS in OxyHb‐induced hippocampal neurons.^[^ ^34^ ^]^ Zhang et al. demonstrated that 2,3,5,4′‐tetrahydroxy‐stilbene‐2‐O‐β‐D‐glucoside (TSG) increased SIRT5 expression to attenuate hepatic steatosis by inhibiting mitochondrial oxidative stress.^[^ ^25^ ^]^ Consistent with these studies, our results demonstrated that SIRT5 improved mitochondrial dysfunction and thus inhibited mitochondrial oxidative stress and inflammation, thereby protecting hepatocytes against AILI. However, one study showed that the SIRT5 inhibitor MC3482 improved mitochondrial function and restored mitochondrial energy homeostasis in free fatty acid‐treated HepG2 cells, which is inconsistent with our results and those of other studies.^[^ ^35^ ^]^ The differential effects of SIRT5 on mitochondria may be due to the fact that SIRT5 regulates mitochondrial function by different mechanisms in different environments or diseases.

Posttranscriptional modifications of proteins include phosphorylation, acetylation, ubiquitination, methylation, and succinylation, and lysine is the most commonly modified residue.^[^ ^36^, ^37^ ^]^ Lysine succinylation is a recently discovered PTM of proteins that plays a key role in the regulation of biological processes, such as fatty acid metabolism, the urea cycle, the tricarboxylic acid cycle, and amino acid degradation.^[^ ^38^, ^39^, ^40^ ^]^ In addition, succinylation has been implicated in a wide range of diseases, including cancer, I/R injury, inflammation and metabolic disorders. Succinylation enhances the conversion of 3‐phosphoglycerate (3‐PG) to 2‐phosphoglycerate (2‐PG) by promoting PGAM1 enzymatic activity, resulting in glycolysis, and proliferation in hepatoma cells.^[^ ^41^ ^]^ Additionally, lysine succinylome analysis revealed 307 differentially modified succinylated sites in 108 proteins, which are involved in various metabolic pathways and cellular processes in human ischemic failing cardiac myofibrils.^[^ ^42^ ^]^ Similarly, SIRT5 plays a pivotal role in liver disease by regulating protein succinylation. SIRT5 KO exacerbates liver I/R injury by increasing the succinylation of peroxiredoxin 3.^[^ ^17^ ^]^ In the present study, we found that the level of protein succinylation in liver tissue and AML12 hepatocytes increased after APAP administration, and that succinylation was further increased in the liver tissue of SIRT5‐KO mice and SIRT5 knockdown AML12 hepatocytes, whereas overexpression of SIRT5 inhibited APAP‐induced succinylation of proteins liver tissue and AML12 hepatocytes. These findings suggest that protein succinylation may play a critical role in the pathogenesis of AILI and that targeted protein succinylation may serve as a novel therapeutic strategy to alleviate APAP‐induced liver injury.

Aldehyde dehydrogenase (ALDH2) is an important detoxifying enzyme secreted by hepatocytes that is located mainly in the mitochondria and protects cells from the harmful effects of endogenous and exogenous toxic substances.^[^ ^43^, ^44^ ^]^ As an essential detoxifying enzyme, ALDH2 efficiently eliminates oxidative stress products and aldehyde metabolites, thereby inhibiting the oxidative stress response, mitigating mitochondrial oxidative stress, and protecting cells from stress‐induced damage.^[^ ^45^, ^46^, ^47^ ^]^ Numerous studies have highlighted the protective role of ALDH2 in mitochondrial oxidative stress‐induced liver diseases, including liver I/R injury, alcoholic liver injury, fatty liver disease, and AILI.^[^ ^48^, ^49^, ^50^, ^51^ ^]^ In this study, we found that ALDH2 interacted with the SIRT5 and that SIRT5 KO increased the succinylation of ALDH2, which inhibited its enzymatic activity. Conversely, SIRT5 overexpression decreased the succinylation of ALDH2, which increased its enzymatic activity. Further LC‒MS/MS analysis revealed a significant increase in succinylation at three lysine residues (K370, K377, K385) in ALDH2 after SIRT5 deletion, and Lys370 (K370) and Lys385 (K385) are highly conserved in different species. By constructing K370 and K385 mutant plasmids, we found that succinylation at the K385 site affected the activity of ALDH2, and cell experiments further verified that SIRT5 alleviated AILI mainly by desuccinylating of ALDH2 at the K385 residue. Moreover, animal experiments confirmed that succinylation at the K385 site abolished the protective effect of ALDH2 against AILI, and desuccinylation of ALDH2 at K385 mediated the protective effect of SIRT5 against AILI. Therefore, the K385 residue may be the dominant functional succinylation site on ALDH2 that is regulated by SIRT5 in AILI. We also confirmed the protective effect of altered ALDH2 enzyme activity against APAP‐induced liver injury using the ALDH2 activator Alda‐1 and the ALDH2 inhibitor Daidzin in SIRT5 knockout mice (Figure S6, Supporting Information).

SIRT5 depletion exacerbates AILI and is accompanied by a significant increase in protein succinylation. In contrast, SIRT5 overexpression attenuated liver injury and reduced protein succinylation levels. Therefore, increasing SIRT5 desuccinylase activity and decreasing protein succinylation may be a promising strategy for ameliorating AILI. We screened natural compounds that could bind to SIRT5 by molecular docking‐based virtual screening, and tested the effect of these compounds on SIRT5 desuccinylase activity. We found that puerarin significantly increased SIRT5 desuccinylase activity and mitigated AILI, indicating that pharmacological activation of SIRT5 desuccinylase activity is a promising strategy for the clinical treatment of AILI. One study has shown that puerarin attenuates APAP‐induced liver injury by activating the Nrf2 signaling pathway,^[^ ^52^ ^]^ and our study also demonstrated the protective effect of puerarin on APAP‐induced liver injury, which is an important addition to the exploration of the mechanism of amelioration of APAP liver injury by puerarin.

In summary, the present study confirmed that SIRT5 acts as a crucial intracellular mediator in the progression of AILI by modulating mitochondrial oxidative stress and that the protective effect of SIRT5 against AILI is dependent on the desuccinylation of ALDH2 at the lysine 385 residue. Therefore, targeting the SIRT5‐ALDH2 axis may be a promising approach for AILI.

## 4 Experimental Section

### Animals

Male C57BL/6 N mice (aged 6–8 weeks and weighing 18–22 g) were obtained from Zhengzhou Huaxin Laboratory Animal Center. SIRT5 global KO mice were generated by Shanghai Nanfang Model Biotechnology Co., Ltd. A liver‐targeted AAV8 system containing a GFP scramble control, SIRT5, ALDH2‐WT, or ALDH2‐385K‐E (designed and synthesized by OBiO Technology Corp., Ltd., Shanghai, China) was injected into the mice via the tail vein at a dose of 1 × 10^12^ vg (200 µL per mouse). The mice were housed in a specific pathogen‐free animal facility with a 12‐h light–dark cycle and had ad libitum access to food and water. AILI was induced by intraperitoneal injection of APAP (Aladdin, Shanghai, China) at the indicated dose following an overnight fasting, and the mice were sacrificed 24 h after APAP administration. All study protocols were approved by the Ethics Committee of the First Affiliated Hospital of Zhengzhou University (2022‐KY‐1402‐001).

### Measurement of Liver Function

Blood samples were collected and centrifuged at 3000 ×g for 5 min, after which the supernatant was collected for analysis. Serum levels of aspartate aminotransferase (AST) and alanine aminotransferase (ALT) were measured using appropriate assay kits according to the manufacturer's instructions (JianChen Bioengineering Institute, Nanjing, China).

### H&E Staining

The liver tissues were fixed in 10% formalin for 48 h, embedded in paraffin, and cut into 5µm thick paraffin sections. After the sections were stained with hematoxylin‐eosin, the histopathological changes in the liver tissues were observed using an optical microscope.

### TUNEL Staining

Paraffin sections were dewaxed, followed by incubation with proteinase K at 37 °C for 20 min. Subsequently, the sections were washed with PBS and incubated with TUNEL detection solution at 37 °C for 10 min. After incubation, the sections were washed three times with PBS for 5 min each time, stained with DAPI, and incubated at room temperature for 10 min. The sections were washed with PBS and sealed with anti‐fluorescence quenching solution before being observed and photographed under a fluorescent microscope.

### Immunohistochemical Staining

Paraffin sections were baked at 60 °C for 2 h, dewaxed by xylene, dehydrated in gradient ethanol, and sections were subjected to thermal antigen repair with 0.01 mol L^−1^ citrate buffer (pH 6.0) and incubated with 3% H_2_O_2_ at room temperature for 10 min to inactivate endogenous enzymes. The sections were incubated with 10% goat serum for 1 h at room temperature, and incubated with the primary antibody overnight at 4 °C. On the next day, the secondary antibody was added and incubated at room temperature for 30 min. The sections were washed three times with PBS before and after the secondary antibody incubation. DAB chromogenic solution was added dropwise, followed by restaining with hematoxylin and sealing with neutral gum, and then observed under a toptical microscope.

### RNA Sequencing and Analysis

Total RNA was extracted from liver tissues using TRIzol reagent (Cwbio, Suzhou, China) according to the manufacturer's protocol. RNA purity and quantification were assessed using a NanoDrop 2000 spectrophotometer (Thermo Scientific, USA). RNA integrity was evaluated using an Agilent 2100 Bioanalyzer (Agilent Technologies, CA, USA). Then, the libraries were constructed using the VAHTS Universal V6 RNA‐seq Library Prep Kit according to the manufacturer's instructions. Transcriptome sequencing and analysis were subsequently conducted by OE Biotech Co., Ltd. (Shanghai, China). Differentially expressed genes were identified using the DESeq (2012) R package.

### Real‐Time Polymerase Chain Reaction (PCR) Analyses

Total RNA was extracted from liver tissues and cells using TRIzol reagent. RNA concentration was measured using a NanoDrop 2000 spectrophotometer (Thermo Scientific, USA), and 1 µg of total RNA was used to synthesize cDNA using a reverse transcription kit (Vazyme,Nanjing, China). cDNA was amplified using SYBR Green qPCR Mix (Vazyme,Nanjing, China) on a qPCR instrument. β‐Actin was used as an internal reference. The 2 ^‐△△CT^ method was used for analysis. The sequences of the primers used for amplification are shown in Table S1 (Supporting Information).

### Transmission Electron Microscopy (TEM)

The liver tissue was cut into 1 mm^3^ pieces. Liver tissue and AML12 hepatocytes were fixed in 4% glutaraldehyde (Servicebio, Wuhan, China) at 4 °C for 2 h, washed with 0.1 mol L^−1^ phosphate buffer, and then fixed in 1% osmium tetroxide. The tissue and cells were dehydrated in gradient acetone and then infiltrated, embedded, and polymerized. The processed specimens were cut into ultrathin sections with a thickness of ≈70 nm, double stained with uranium lead, and subsequently observed under a transmission electron microscope.

### Western Blot

Protein was extracted from liver tissue and cells by using RIPA lysis buffer (Solarbio, Beijing, China), and the protein concentration was determined using a bicinchoninic acid kit (Solarbio, Beijing, China). The proteins were separated by SDS‐PAGE, transferred to PVDF membranes, blocked with 5% skim milk at room temperature for 1 h, and incubated with primary antibodies overnight at 4 °C, followed by incubation with goat anti‐rabbit or goat anti‐mouse secondary antibody at room temperature for 1 h. Protein bands were detected using an enhanced chemiluminescent reagent kit (Biosharp, Hefei, China), and the signals were visualized using an ImageQuant™ System (Cytiva, America). The antibodies used for immunoblotting are shown in Table S2 (Supporting Information).

### Cell Culture and APAP Administration

AML12 mouse hepatocytes were obtained from Procell Biotechnology Co. (Wuhan, China). The cells were cultured in DMEM/F12 medium supplemented with 10% fetal bovine serum (Gibco, Carlsbad, CA, USA), 1×10^5^ U L^−1^ penicillin and 100 mg L^−1^ streptomycin (Solarbio, Beijing, China) in a constant temperature incubator at 37 °C with 5% CO_2_. APAP was dissolved in culture medium at 37 °C and added to the cells after being filtered through a 0.22 µm filter.

### Plasmid and Cell Transfection

The ALDH2 and SIRT5 expression plasmids, as well as the ALDH2 mutant plasmid, were synthesized by OBiO Technology Corp., Ltd. (Shanghai, China). The plasmids were transfected into HEK 293T cells or AML12 hepatocytes with Lipo2000 transfection reagent according to the manufacturer's instructions (Biosharp, Hefei, China). To establish stable SIRT5 knockdown and overexpression cell lines, a SIRT5 lentiviral plasmid and lentiviral vectors were transfected into HEK 293T to generate lentiviral particles, which were subsequently transfected into AML12 hepatocytes, and the cells were selected using medium containing 2 µg mL^−1^ puromycin (Gibco, Carlsbad, CA, USA) for 7 days. The sequences of the lentiviral plasmids used for SIRT5 knockdown are listed in Table S3 (Supporting Information).

### Measurement of Cell Viability

AML12 hepatocytes were seeded into 96‐well plates (100 µL per well) at a density of 0.5 × 10^4^ cells mL^−1^. After 12 h, APAP was added to the wells. After incubation for 24 h incubation, cell viability was determined using a cell counting kit‐8 according to the manufacturer's instructions (Beyotime, Shanghai, China).

### Enzyme‐Linked Immunosorbent Assay (ELISA)

Serum concentrations of *IL‐1β*, *IL‐6*, and *TNF‐α* were detected by ELISA kits according to the manufacturer's protocol (Proteintech, Wuhan, China).

### Detection of MDA, SOD, GSH, and ROS

To examine MDA, SOD, and GSH, mouse liver tissue was homogenized with extraction solution at the following ratio: weight (g):volume (mL) = 1:9. The supernatant was collected by centrifugation at 8000 r min^−1^ at 4 °C for 10 min. Protein concentration was determined by a BCA detection kit, and the levels of MDA, SOD, and GSH in liver tissues were detected in accordance with the instructions (Solarbio, Beijing, China). Staining with the fluorescent probe dihydroethidium (DHE) was used to measure ROS concentrations in tissues. Briefly, OTC‐embedded liver tissues were cut into 10 µm‐thick slices, stained with 10 µM DHE (Biosharp, Hefei, China), incubated at room temperature for 1 h in the dark, and then stained with DAPI (Biosharp, Hefei, China) for 10 min. Finally, the sections were observed under a fluorescence microscope.

### JC‐1 and MitoSOX Staining

JC‐1 dye was used to measure mitochondrial membrane potential in AML12 hepatocytes. After APAP administration, the cells were incubated with JC‐1 (10 µg mL^−1^) for 20 min at 37 °C, washed two times with staining buffer, and then observed under a fluorescence microscope. To measure mitochondrial ROS levels, cells were incubated with 5 µM MitoSOX working solution (Thermo Fisher Scientific) for 10 min at 37 °C in the dark. Then, the cells were washed three times with warm HBSS buffer and visualized by using a fluorescence microscope.

### Liquid Chromatography–Tandem Mass Spectrometry (LC‒MS/ MS) Analysis of Peptide Succinylation

LC‒MS/ MS analysis of peptide succinylation products was performed and analyzed by PTM BIO (Hanzhou, China). The detailed methods are shown in the Supporting Information.

### Immunoprecipitation and Coimmunoprecipitation (Co‐IP)

Cells and liver tissues were lysed using IP lysis buffer (Beyotime, Shanghai, China) and centrifuged at 12 000 × g at 4 °C for 10 min to obtain protein lysates. The lysates were incubated overnight at 4 °C with protein A+G agarose beads (Beyotime, Shanghai, China) and the corresponding primary antibody. The beads were washed three times with IP lysis buffer and boiled with 2 × loading buffer at 95 °C for 5 min to obtain the proteins. Finally, the proteins were subjected to immunoblot analysis.

### Immunofluorescence Staining

AML12 hepatocytes were fixed in 4% paraformaldehyde and incubated overnight at 4 °C with primary antibodies against SIRT5 and ALDH2. After being washed three times with PBS, the cells were incubated with goat anti‐rabbit and goat anti‐mouse secondary antibodies at 37 °C for 1 h. Subsequently, the nuclei were stained with DAPI for 10 min at room temperature. Images were obtained under a fluorescence microscope.

Paraffin sections were baked at 60 °C for 2 h, dewaxed by xylene, dehydrated in gradient ethanol, and sections were subjected to thermal antigen repair with 0.01 mol L^−1^ citrate buffer (pH 6.0). The sections were incubated with 10% goat serum for 1 h at room temperature, and antibodies incubation, DAPI staining, and photography were the same as for cells.

### Molecular Docking‐Based Virtual Screening

Molecular docking‐based virtual screening was used to screen for SIRT5 activators, and the detailed methods are shown in the Supporting Information.

### Molecular Docking and Molecular Dynamics Simulation

Molecular docking and molecular dynamics simulations were used to determine the interaction between Puerarin and SIRT5, and the detailed methods are shown in the Supporting Information.

Measurement of ALDH2 and SIRT5 desuccinylase activity: ALDH2 activity was measured using an ALDH2 activity assay kit according to the manufacturer's instructions (Solarbio, Beijing, China). SIRT5 desuccinylase activity was measured using a luorometric assay kit according to the manufacturer's instructions (BPS bioscience, San Diego, USA).

### Statistical Analysis

All the statistical analyses were performed using SPSS 2 2.0 statistical software. The results are expressed as the mean ± SD. A two‐tailed Student *t* test was used for comparisons between two groups. One‐way ANOVA followed by the Tukey test was used for comparisons between multiple groups. *P* < 0.05 was considered to indicate statistical significance.

## Conflict of Interest

The authors declare no conflict of interest.

## Author contributions

Q.Y., J.Z., and J.L. contributed equally to this work. Q.Y., J.Z., J.L., and S.C. performed experiments, analyzed data, and prepared the manuscript. Y.S., J.P., C.M., M.C., Q.H., H.W., H.L., B.C., and Y.Z. performed experiments and collected data. W.G., C.Z., and S.C. oversaw the project and proofread the manuscript. Q.Y. and S.C. designed the project, oversaw the experiments, and prepared the manuscript. All authors read and approved the final manuscript.

## Supporting information

Supporting Information

## Data Availability

The data that support the findings of this study are available from the corresponding author upon reasonable request.
