# Supplementary material for: Sirtuin 5‐Mediated Desuccinylation of ALDH2 Alleviates Mitochondrial Oxidative Stress Following Acetaminophen‐Induced Acute Liver Injury
Source: Adv Sci (Weinh). 2024 Aug 19;11(39):2402710. doi: 10.1002/advs.202402710 (PMC11497042; doi:10.1002/advs.202402710)

**Supporting Information**

Sirtuin 5-mediated desuccinylation of ALDH2 alleviates mitochondrial oxidative stress following acetaminophen-induced acute liver injury

Qiwen Yu, Jiakai Zhang, Jiye Li, Yaodong Song, Jie Pan, Chaopeng Mei, Mengwei Cui, Qianqian He, Haifeng Wang, Huihui Li, Bo Cheng, Yan Zhang, Wenzhi Guo*, Changju Zhu*, and Sanyang Chen*

**Supplementary Materials and Methods**

**1 Animals**

SIRT5 KO mice were treated intraperitoneally 30 min before APAP administration with either 20 mg/kg ALDH2 agonist Alda-1 (MedChemExpress**,** Shanghai, China) or 100 mg/kg ALDH2 inhibitor Daidzin (MedChemExpress**,** Shanghai, China). Both Alda-1 and Daidzin were dissolved in 0.1% DMSO (Solarbio, Beijing, China). AILI was induced by intraperitoneal injection of APAP ([Aladdin](https://www.baidu.com/other.php?url=Kf0000aekflRNvwhJ6C682eY_3yAI3a2w7xzN0wXC4r-ZRvgF2-3D-477harJm8iwMF5JbmjqMH8xnngkJvL40OQfHAXcFQrorE71fJo_AKm-E5kc5VBAt9CcORku609CLfGJZ9v0kUakLGLPk620JVMvkG9wd8LHfrrGYVgfZ82tCw8Vh0FeL4qQrJeffH8NN3fjW_7p32WfcQL7xi5OxX18Pjg.7R_NR2Ar5Od663rj6thpQPd13_lRf2N9h9m34PLWm0.TLFWgv-b5HDkrfK1ThPGujYknHb0THY0IAYq_2Q0oUp90ZN1ugFxIZ-suHYs0A7bgLw4TARqP6KLULFb5UaBs2vvCfKzmLmqn0KdThkxpyfqnHRdPjTkPHmznsKVINqGujYkPHTdP16LP0KVgv-b5HDzrH0srHcs0AdYTAkxpyfqnHc3nWm0TZuxpyfqn0KGuAnqiDF70ZKGujYkP0KWpyfqn1cz0APzm1Y1P1mYPf&ck=4500.12.26659.368.162.378.149.425&dt=1701524081&wd=%E9%98%BF%E6%8B%89%E4%B8%81&tpl=tpl_12826_33676_0&l=1554715623&ai=0_54736373_1_0&us=linkVersion=1&compPath=10036.0-10032.0&label=%E4%B8%BB%E6%A0%87%E9%A2%98&linkType=&linkText=%E9%98%BF%E6%8B%89%E4%B8%81aladdin-%E4%B8%93%E4%B8%9A%E7%A7%91%E7%A0%94%E8%AF%95%E5%89%82%E7%94%9F%E4%BA%A7%E5%95%86), Shanghai, China) at 400mg/kg after an overnight fast, and the mice were sacrificed 24 h after APAP administration.

Ten compounds with the lowest affinity energy were purchased from MedChemExpress LLC (Shanghai, China), information and methods of administration for ten compounds are listed in Supplementary Table 4. APAP treatment was performed after the last administration and blood and tissue samples were collected 24 hours later for testing.

2 Liquid chromatography–tandem mass spectrometry (LC‒MS/ MS) analysis of peptide succinylation

**2.1 Protein Extraction**

100 mg sample was grinded with liquid nitrogen into cell powder and then transferred to a 5-mL centrifuge tube. After that, four volumes of lysis buffer (8 M urea, 1% protease inhibitor cocktail) w**ere** added to the cell powder, followed by sonication three times on ice using a high intensity ultrasonic processor (Scientz). The remaining debris was removed by centrifugation at 12,000 g at 4 °C for 10 min. Finally, the supernatant was collected and the protein concentration was determined with BCA kit according to the manufacturer’s instructions.

**2.2 Trypsin Digestion**

For digestion, the protein solution was reduced with 5 mM dithiothreitol for 30 min at 56 °C and alkylated with 11 mM iodoacetamide for 15 min at room temperature in darkness. The protein sample was then diluted by adding 100 mM TEAB to urea concentration less than 2 M. Finally, trypsin was added at 1:50 trypsin-to-protein mass ratio for the first digestion overnight and 1:100 trypsin-to-protein mass ratio for a second 4 h-digestion. Finally, the peptides were desalted by C18 SPE column.

**2.3 Affinity Enrichment**

**Pan-antibody-based PTM enrichment:** To enrich modified peptides, tryptic peptides dissolved in NETN buffer (100 mM NaCl, 1 mM EDTA, 50 mM Tris-HCl, 0.5% NP-40, pH 8.0) were incubated with pre-washed antibody beads (Lot number PTM402, PTM Bio) at 4°C overnight with gentle shaking. Then the beads were washed for four times with NETN buffer and twice with H_2_O. The bound peptides were eluted from the beads with 0.1% trifluoroacetic acid. Finally, the eluted fractions were combined and vacuum-dried. For LC-MS/MS analysis, the resulting peptides were desalted with C18 ZipTips (Millipore) according to the manufacturer’s instructions.

**2.4 4D Mass Spectrometer**

The tryptic peptides were dissolved in solvent A (0.1% formic acid, 2% acetonitrile/in water), directly loaded onto a home-made reversed-phase analytical column (25-cm length, 75/100 μm i.d.). Peptides were separated with a gradient from 6% to 24% solvent B (0.1% formic acid in acetonitrile) over 70 min, 24% to 35% in 14 min and climbing to 80% in 3 min then holding at 80% for the last 3 min, all at a constant flow rate of 450 nL/min on a nanoElute UHPLC system (Bruker Daltonics).

The peptides were subjected to capillary source followed by the timsTOF Pro (Bruker Daltonics) mass spectrometry. The electrospray voltage applied was 1.60 kV. Precursors and fragments were analyzed at the TOF detector, with a MS/MS scan range from 100 to 1700 m/z. The timsTOF Pro was operated in parallel accumulation serial fragmentation (PASEF) mode. Precursors with charge states 0 to 5 were selected for fragmentation, and 10 PASEF-MS/MS scans were acquired per cycle. The dynamic exclusion was set to 30 s.

**2.5 Database Search**

The resulting MS/MS data were processed using MaxQuant search engine (v.1.6.15.0). Tandem mass spectra were searched against the human SwissProt database (20422 entries) concatenated with reverse decoy database. Trypsin/P was specified as cleavage enzyme allowing up to 2 missing cleavages. The mass tolerance for precursor ions was set as 20 ppm in first search and 5 ppm in main search, and the mass tolerance for fragment ions was set as 0.02 Da. Carbamidomethyl on Cys was specified as fixed modification, and acetylation on protein N-terminal, oxidation on methionine and succinylation of lysine were specified as variable modifications. False discovery rate (FDR) was adjusted to < 1%.

Liquid chromatography-tandem mass spectrometry (LC-MS/MS) datas are available in the iProX database (<https://www.iprox.cn/>), the Project ID is PXD050121.

3 Molecular Docking-Based Virtual Screening

For virtual screening of SIRT5 activators, approximately 2100 natural products from commercially available sources were chosen to establish a screening library. The structural formula (SDF format) of the compounds was downloaded from the PubChem database and converted into PDB format. The crystal structure of the SIRT5 (AF-Q8K2C6) was downloaded from the AlphaFold Protein Structure Database (https://alphafold.ebi.ac.uk/). Hydrogen atoms were added, and crystallographic water molecules were deleted. SAM and the SIRT5 were separated by AutoDock Vina 1.1.2. The library compound file in PDB format was imported into AutoDock Vina. Atomic charges were added, atomic types were assigned and all flexible bonds were rotatable by default. The library compounds were then docked into the SAM binding site of SIRT5 by AutoDock Vina 1.1.2. Compounds with affinity (kcal/mol) (free energy ≤−10 kcal/mol) and readily available commercial sources were selected for biological evaluation using the SIRT5 desuccinylase in vitro activity assay.

4 Molecular docking and Molecular Dynamics Simulation

Download the 3D structure of SIRT5 from Protein Data Bank database and Puerarin's 3D structure from ZINC database, and use AutoDock Tool to remove water, hydrogenate, minimize energy, etc. Autodock Vina 1.1.2 was used for molecular docking and the results were calculated. The results were visualized with PyMOL.

We utilized Amber20 for conducting molecular dynamics simulations on protein-small molecule ligand complexes. The Puerarin were treated with the general AMBER force field (gaff), and the SIRT5 proteins were processed using the ff14SB force field. The amino acid residues of the SIRT5 proteins were assigned their default protonation states in Amber20 and were hydrogenated using the tleap module. Structure optimization and electrostatic potential calculations for Puerarin were performed using the Gaussian09 software package based on B3LYP/6-31G, and partial charges were fitted using the RESP method in the Antechamber module. Molecular mechanics optimization and molecular dynamics simulations of the complexes were executed using the sander or pmemd programs in Amber20. After balancing ions to maintain neutrality, the entire system was placed in a rectangular water box of TIP3P, extending to a three-dimensional space of 12Å×12Å×12Å around the solute molecule. Prior to MD simulation, the system underwent two steps of energy optimization. Initially, the solute molecules were fixed for 10,000 steps of energy optimization, including 5,000 steps of steepest descent and 500 steps of conjugate gradient; subsequently, constraints were released for another 10,000 steps of optimization, also involving 5,000 steps of steepest descent and 5,000 steps of conjugate gradient. During MD simulations, the Particle mesh Ewald (PME) method was employed to handle long-range electrostatic interactions, and the SHAKE method was used to constrain all bonds connected to hydrogen atoms, with a time step set at 2 fs. Non-bonded interactions were subjected to a cut-off value of 10Å. The entire system was heated from 0 K to 300 K in 100 ps under constant volume using constraints, followed by equilibrating solvent density under constant temperature and pressure conditions (T = 300 K, P = 1 atm), and finally sampled at constant pressure for 100 ns, saving one frame (conformation) per ps for subsequent analysis.

5 Measurement SIRT5 desuccinylase activity

The desuccinylation activity of SIRT5 was measured 24 h after APAP treatment with a fluorometric assay kit (#50085, BPS bioscience, San Diego, USA). Fresh liver tissue samples were mechanically homogenised and ultrasonically disrupted in RIPA lysis buffer (Solarbio, Beijing, China). Tissue lysates were centrifuged at 20,000 g for 15 min at 4 °C. The supernatants were collected and the protein concentration was detected using a BCA protein concentration determination assay kit (Solarbio, Beijing, China). The desuccinylase reaction was initiated by adding 25 μL of the master mixture (5 μL SIRT substrate (100μM), 0.5 the protein NAD+, 5 μL BSA (1mg/ml), 14.5 μL SIRT assay buffer) to the designated test wells and blank wells on a black microtiter plate. Subsequently, add 5 µL (10 μg/the protein) samples and 20 μL of diluted SIRT5 enzyme to the designated test well, 20 μL SIRT assay buffer and 5 μL inhibitor buffer to the designateg blank well. Finally, adding 50 μL of undiluted SIRT assay developer (2×) to each well and incubated at 25 °C for 15 minites. Then, the fluorescence was read with excitation at a wavelength in the range of 350-380 nm and detection of emitted light in the range of 440-460 nm at 25 °C on a microplate reader.

**Supplementary Figures**

**Supplementary Figure 1**

Supplementary Figure 1. Cell activity of AML12 hepatocytes in different groups indicated 24 h after APAP treatment (n=**6**)**.** All data are presented as the mean ± SD. Levels of statistical significance are indicated as ***P* < 0.01. One-way ANOVA with Tukey test analysis was used for statistical analysis.

**Supplementary Figure 2**


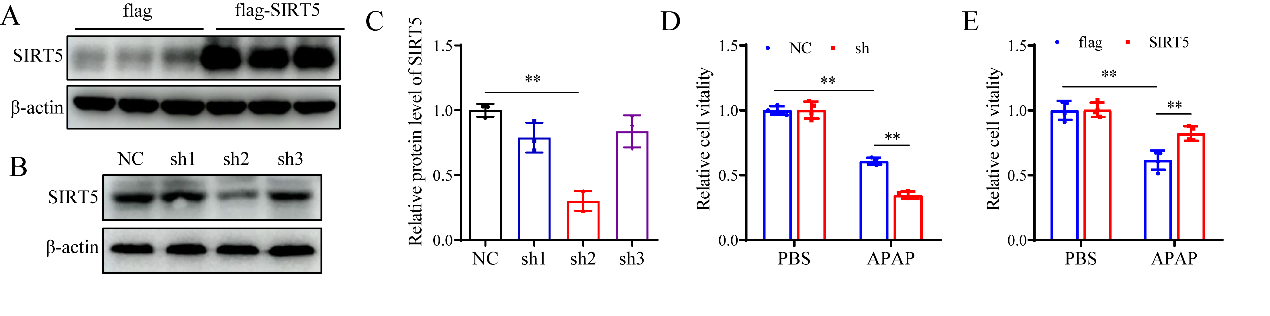


Supplementary Figure 2. SIRT5 ameliorates APAP-induced hepatotoxicity *in vitro*. (A) SIRT5 protein expression in stable overexpression AML12 hepatocytes (n=3). (B, C) SIRT5 protein expression and statistical analysis in stable knockdown AML12 hepatocytes (n=4). (D, E) Cell activity of AML12 hepatocytes in different groups indicated 24 h after APAP treatment (n=5). All data are presented as the mean ± SD. Levels of statistical significance are indicated as ***P* < 0.01; ns, non-significant. One-way ANOVA with Tukey test analysis and Student t test were used for statistical analysis.

**Supplementary Figure 3**


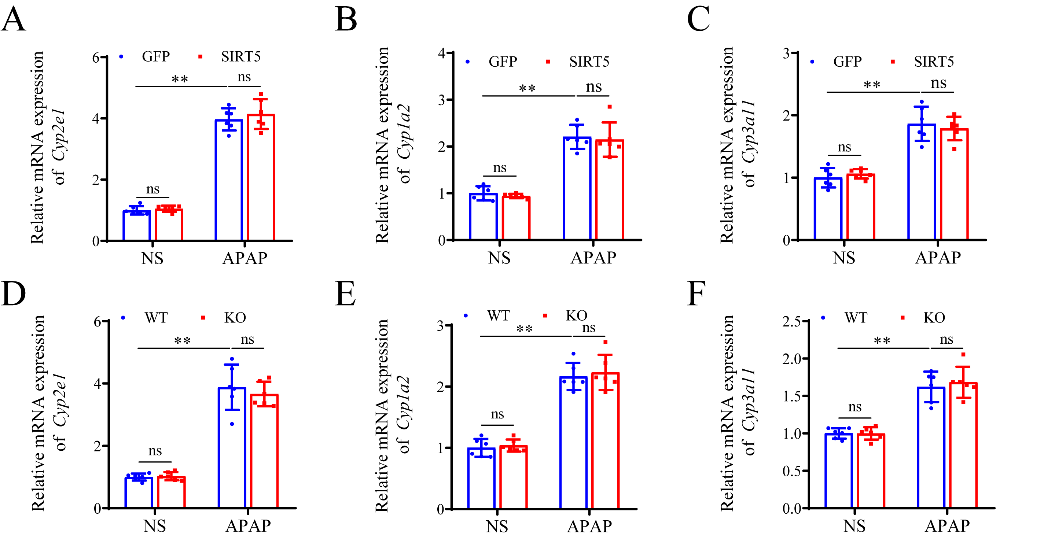


Supplementary Figure 3. SIRT5 has no significant effect on the expression of Cyp2e1, Cyp1a2 and Cyp3a11. (A-C) mRNA expression of the cytochrome P450 enzymes Cyp2e1, Cyp1a2 and Cyp3a11 in liver tissues of AAV-GFP and AAV-SIRT5 mice treated with APAP for 24 h (n=6). (D-**F**) mRNA expression of the cytochrome P450 enzymes Cyp2e1, Cyp1a2 and Cyp3a11 in liver tissues of WT and SIRT5 KO mice treated with APAP for 24 h (n=6). Data are presented as the mean ± SD.^**^*P* < 0.01; ns, non-significant. One-way ANOVA with Tukey test analysis and Student t test were used for statistical analysis.

**Supplementary Figure 4**


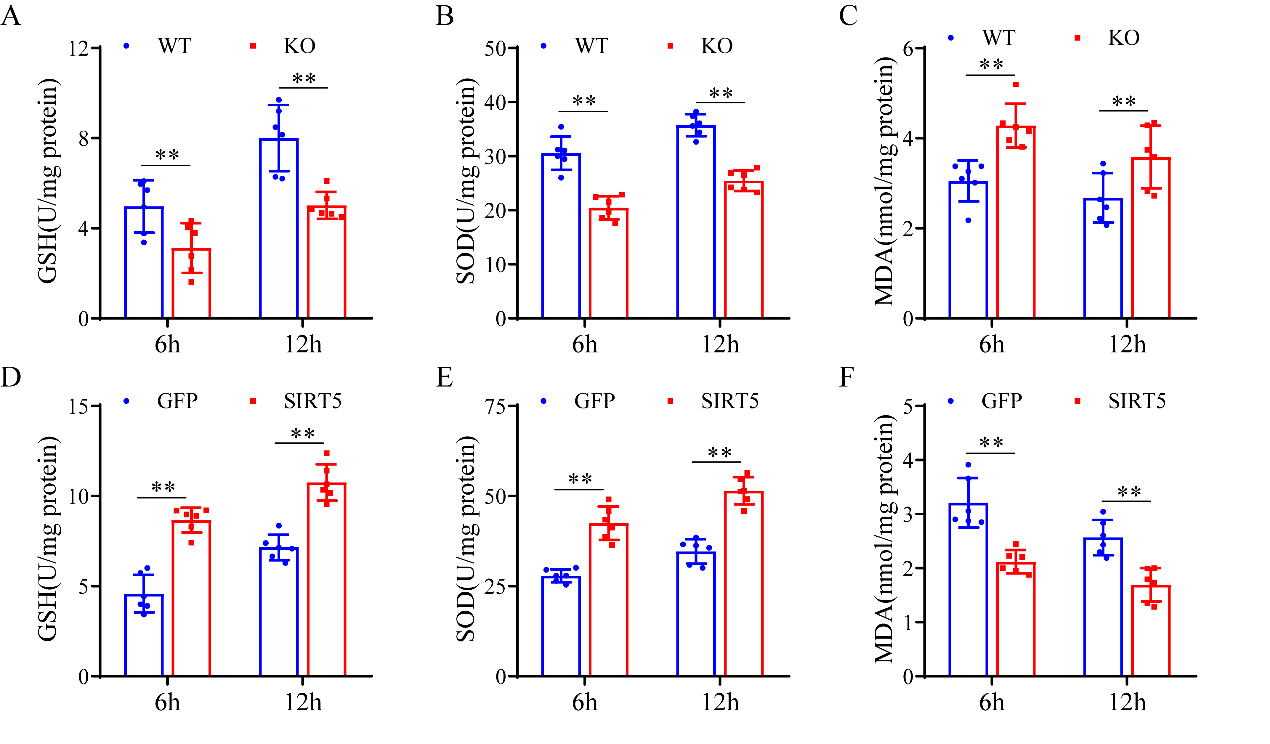


Supplementary Figure 4. SIRT5 inhibits APAP-induced mitochondrial oxidative stress in AILI. (A-C) Liver GSH, MDA and SOD level in WT and SIRT5-KO mice treated with APAP for 6 and 12 h (n=6). (D-E) Liver GSH, MDA and SOD level in AAV-GFP and AAV-SIRT5 mice treated with APAP for 6 and 12 h (n=6). All data are presented as the mean ± SD. Levels of statistical significance are indicated as ^**^*P* < 0.01; ns, not significant. One-way ANOVA with Tukey test analysis and Student t test were used for statistical analysis.

**Supplementary Figure 5**


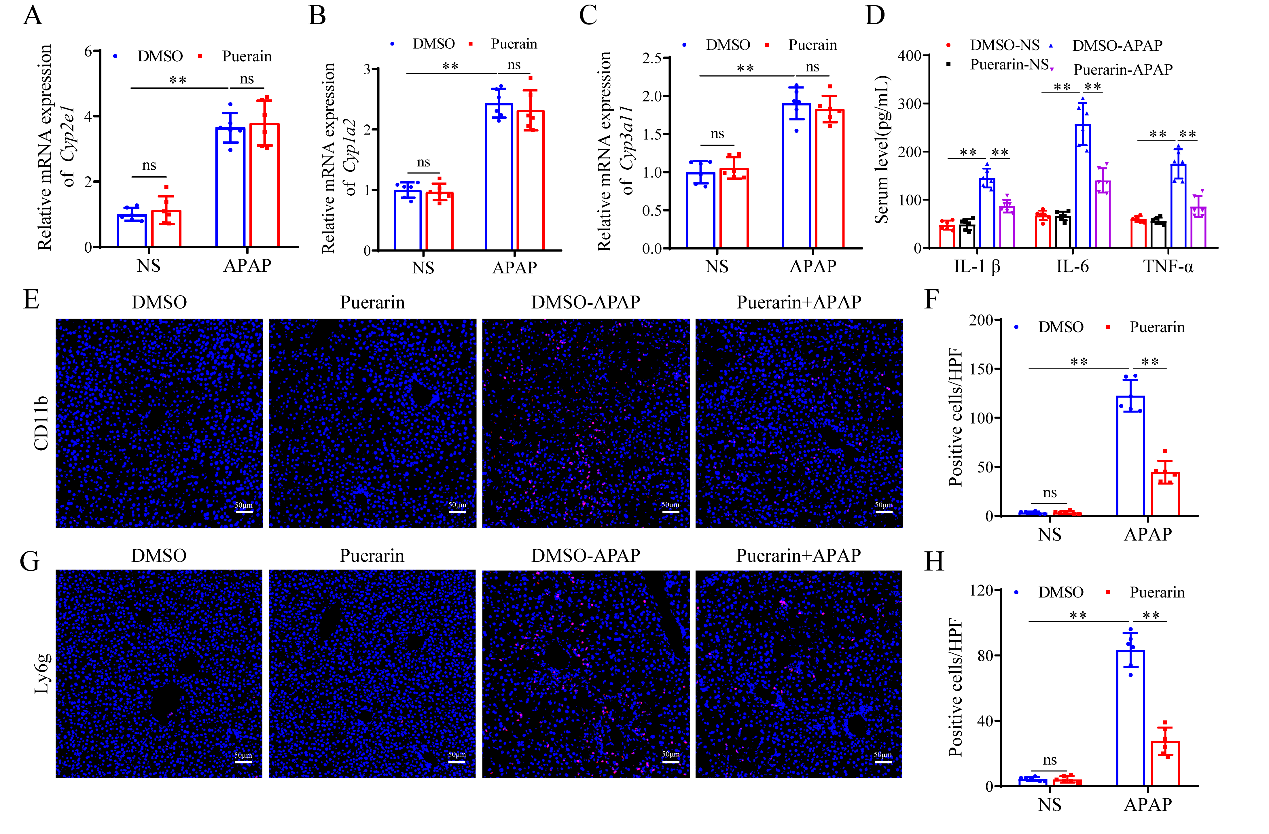


Supplementary Figure 5. Puerarin attenuates inflammation and has no significant effect on the expression of Cyp2e1, Cyp1a2 and Cyp1a2 after APAP administration. (A-C) mRNA expression of the cytochrome P450 enzymes Cyp2e1, Cyp1a2 and Cyp3a11 in liver tissue of DMSO and puerarin treated mice at 24 h after APAP treatment (n=6). (D) Serum levels of inflammatory cytokines TNF-α, IL-1β and IL-6 in mice described in DMSO and puerarin treated mice at 24 h after APAP treatment (n=6). (E-H) Immunofluorescence staining of CD11-b and Ly6g-positive inflammatory cells (red) and statistics of liver tissue in mice described in DMSO and puerarin treated mice at 24 h after APAP treatment (n=6). All Data are presented as the mean ± SD. Levels of statistical significance are indicated as ^*^*P* < 0.05, ^**^*P* < 0.01; non-significant. One-way ANOVA with Tukey test analysis and Student t test were used for statistical analysis.

**Supplementary Figure 6**


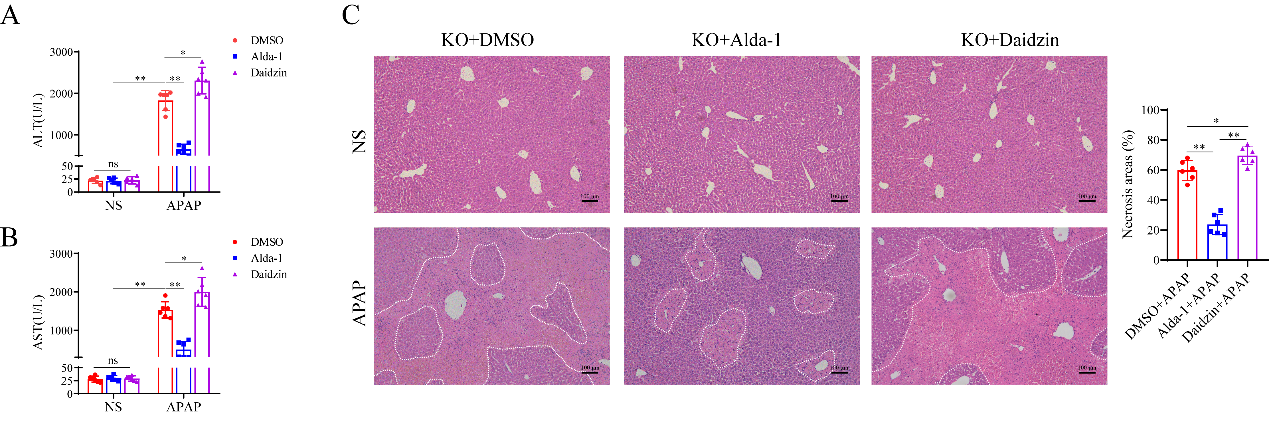


Supplementary Figure 6. Alda-1 pretreatment ameliorates APAP-induced liver hepatotoxicity. (A, B) Serum levels of ALT and AST in each group of mice treated with APAP for 24 h (n=6). (C) H&E staining and necrotic area statistics of liver tissues in each group of mice treated with APAP for 24 h (n=6). Data are presented as the mean ± SD.^**^*P* < 0.01; ns, non-significant. One-way ANOVA with Tukey test analysis and Student t test were used for statistical analysis.

**Supplementary Tables**

Supplementary Table 1. Primers for real-time PCR detection

| Primer | Primer sequence |
| --- | --- |
| Sirt5 F | GCCACCGACAGATTCAGGTT |
| Sirt5 R | CCACAGGGCGGTTAAGAAGT |
| Cyp2e1 F | AGGCTGTCAAGGAGGTGCTACT |
| Cyp2e1 R | AAAACCTCCGCACGTCCTTCCA |
| Cyp1a2 F | CATCACAAGTGCCCTGTTCAAGC |
| Cyp1a2R | AATGCTCCAGGTGATGGCTGTG |
| Cyp3a11F | GGAAGCATTGAGGAGGATCACA |
| Cyp3a11 R | AGGTCCATCCCTGCTTGTTT |
| β-actin F | TGAGCTGCGTTTTACACCCT |
| β-actin R | CGCCTTCACCGTTCCAGTTT |

Supplementary Table 2. Antibodis information

| Antibody | Supplier | Cat No. | Concentration |
| --- | --- | --- | --- |
| SIRT5 | Proteintech | 15122-1-AP | 1:2000 for WB |
| p-p65 | CST | 3033 | 1:1000 for WB |
| p65 | Proteintech | 10745-1-AP | 1:1000 for WB |
| p-IKKβ | CST | 2697 | 1:1000 for WB |
| IKKβ | Proteintech | 15649-1-AP | 1:600 for WB |
| p-IkBα | CST | 2859 | 1:1000 for WB |
| ALDH2 | Proteintech | 15310-1-AP | 1:5000 for WB; 2 μg for IP |
| Anti-Succinyllysine | PTM BIO | PTM-419 | 1:1000 for WB |
| Anti-HA Tag | HUABIO | 0906-1 | 1:5000 for WB;2 μg for IP |
| Anti-flag Tag | Proteintech | 80010-1-RR | 1:5000 for WB;2 μg for IP |
| β-actin | Proteintech | 81115-1-RR | 1:5000 for WB |
| CD11b | Servicebio | GB115689 | 1:200 for IF |
| Ly6g | Servicebio | GB11229 | 1:200 for IF |

Supplementary Table 3. SIRT5 knockdown lentivirus plasmid sequence

| Plasmid | Sequence |
| --- | --- |
| NC | CGAGTAGGTGCAGAGTTAGCT |
| Sh-1 | CCAGTTGTGTTGTAGACGAAA |
| Sh-2 | CGACAGATTCAGGTTTCATTT |
| sh-3 | CGAGAACTATAGGAGTCCGAT |

Supplementary Table 4 Information and methods of administration for ten compounds

| Compd. | Cat.No. | Administration |
| --- | --- | --- |
| Bilobetin | HY-N2118 | Intraperitoneal injection,12 mg/kg/day,7 days |
| Morellic acid | HY-N4094 | Intravenously, 2 mg/kg/day, 5 days |
| Amentoflavone | HY-N0662 | Intraperitoneal injection, 40 mg/kg/day, 7 days |
| (+)-Bicuculline | HY-N0219 | Intraperitoneal injection, 0.25 mg/kg/day, 5 days |
| Strictosamide | HY-N1198 | Intraperitoneal injection, 40 mg/kg/day, 5 days |
| Irinotecan Hydrochloride | HY-16562A | Intraperitoneal injection, 5 mg/kg/day, 5 days |
| Ginkgetin | HY-N0889 | Intraperitoneal injection, 100 mg/kg/day, 4 days |
| Pueraria glycoside | [HY-N1980](https://www.medchemexpress.cn/3-hydroxypuerarin.html) | Intragastrically, 100 mg/kg/day, 10 days |
| Irinotecan hydrochloride trihydrate | HY-16568 | Intraperitoneal injection, 5 mg/kg/day, 5 days |
| Puerarin | HY-N0145 | Intragastrically, 100 mg/kg/day, 10 days |

**Uncropped Western blots films**


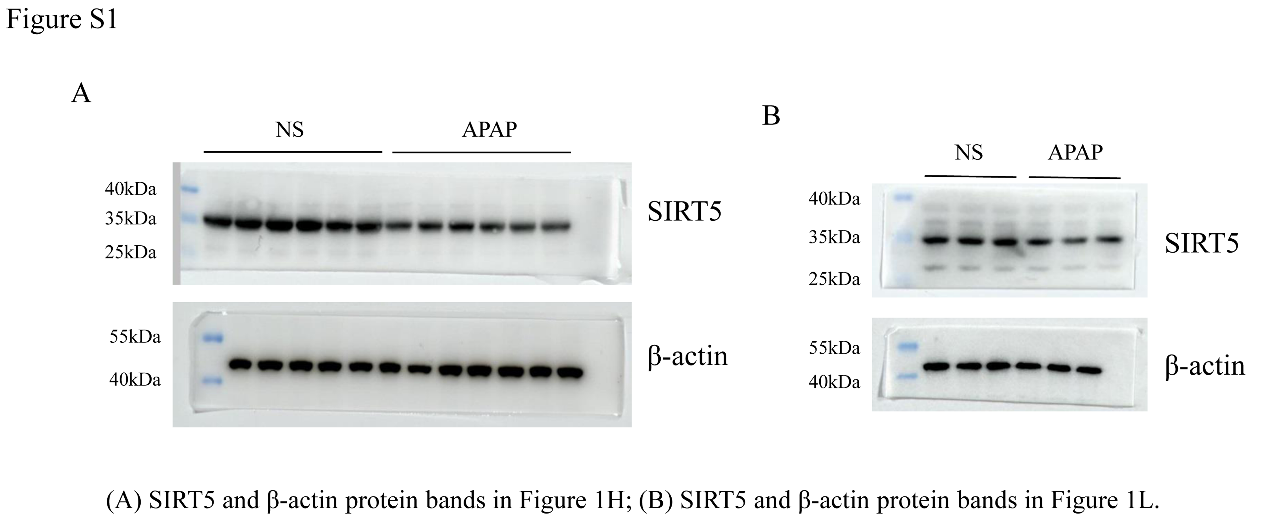


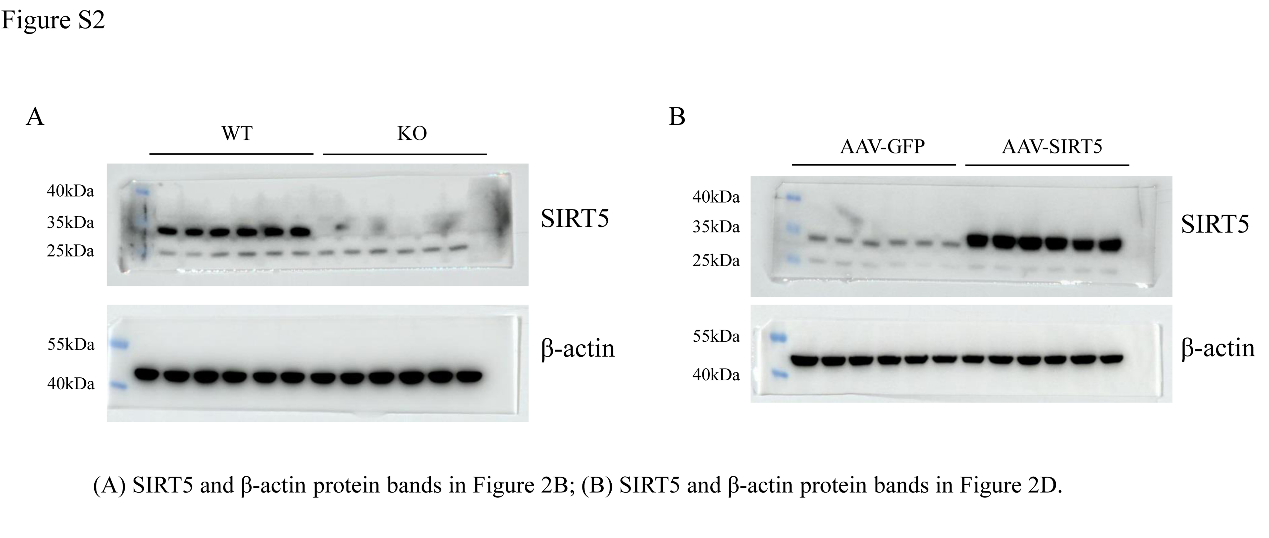

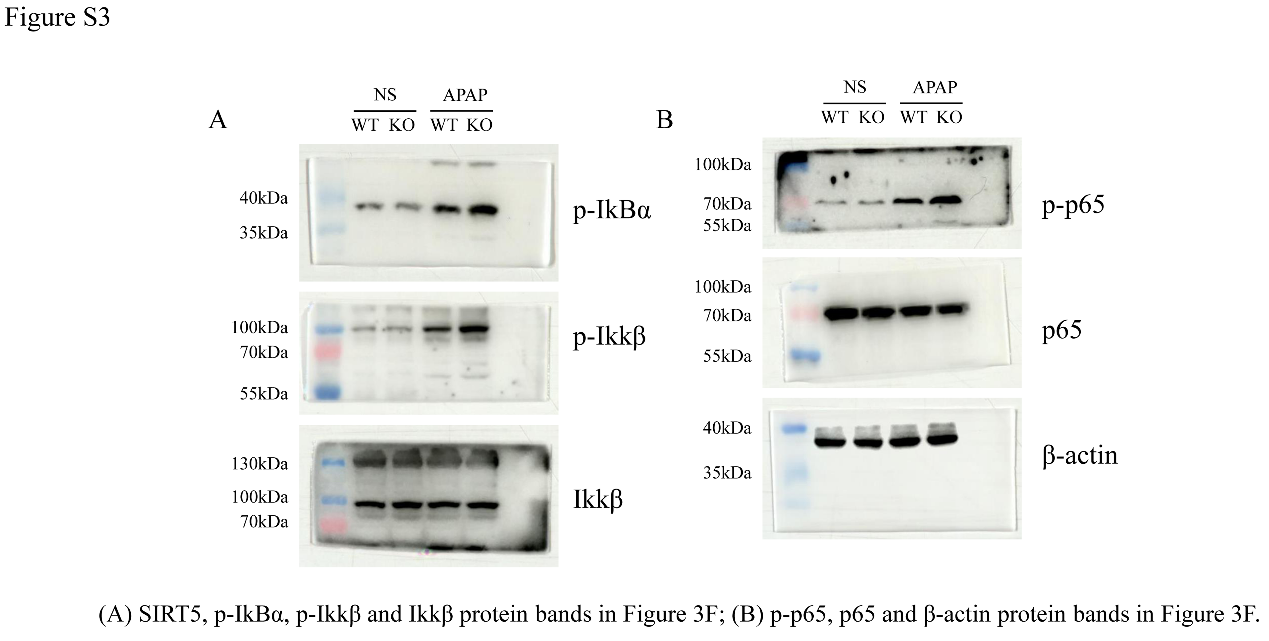


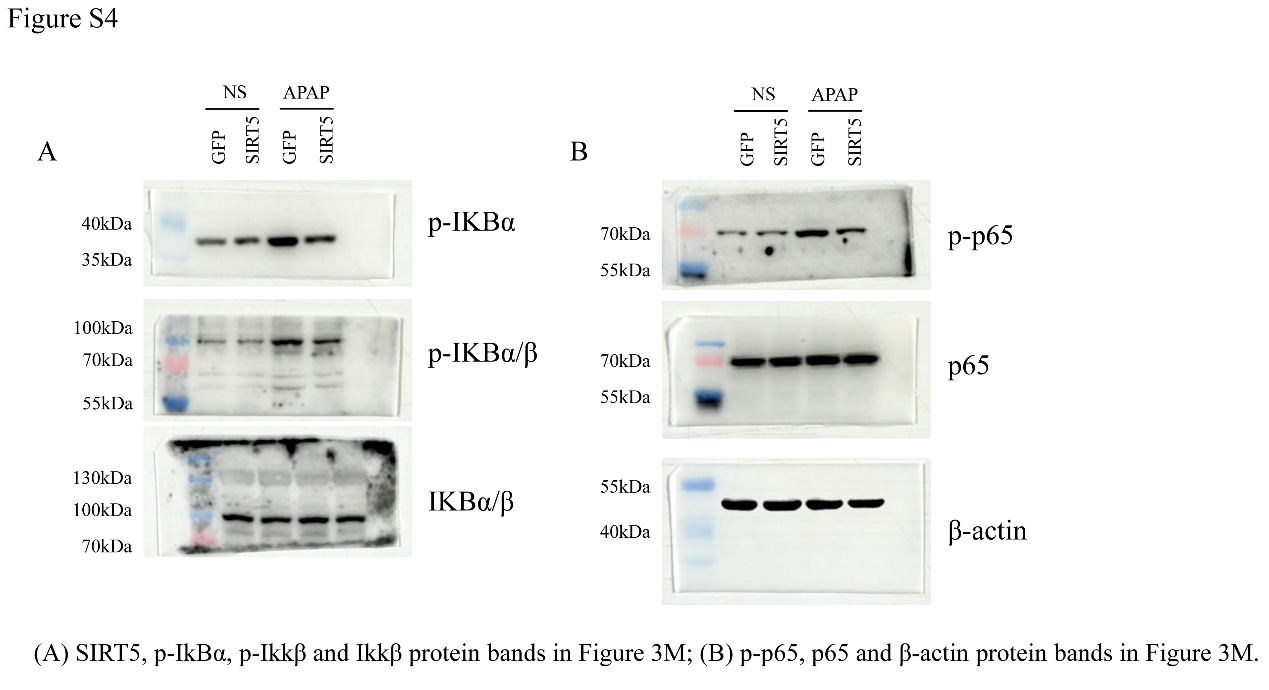

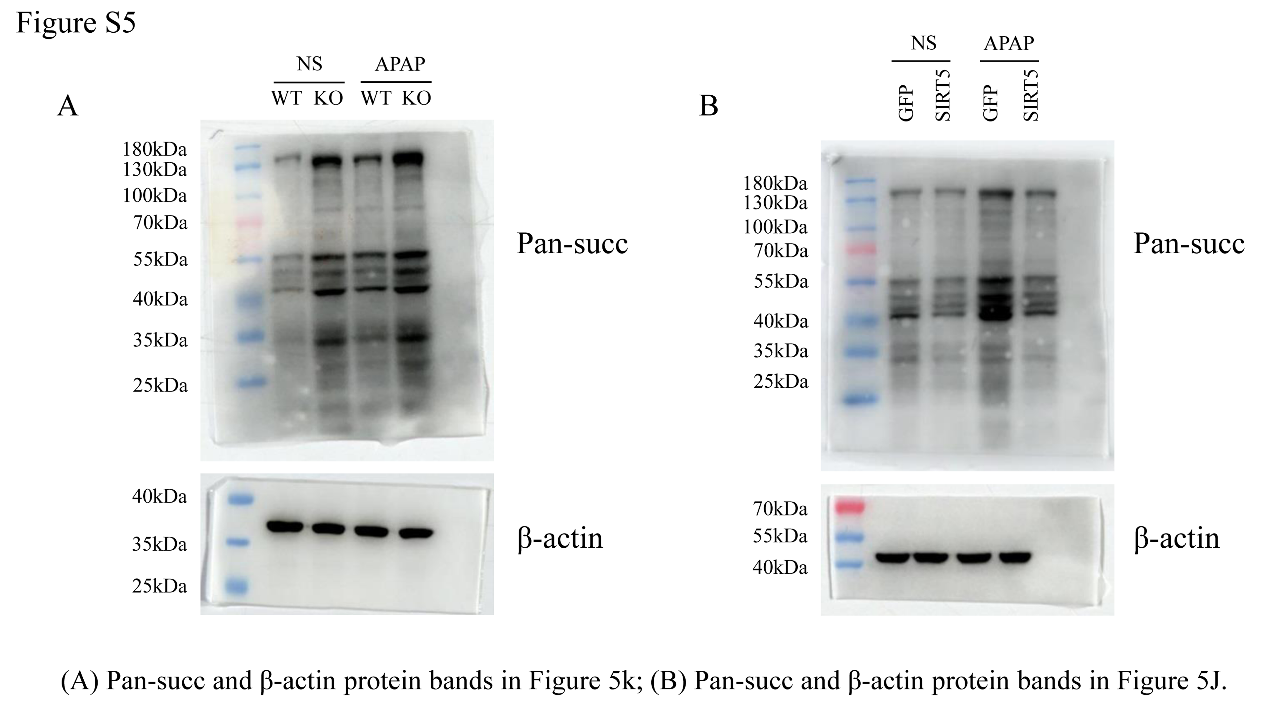


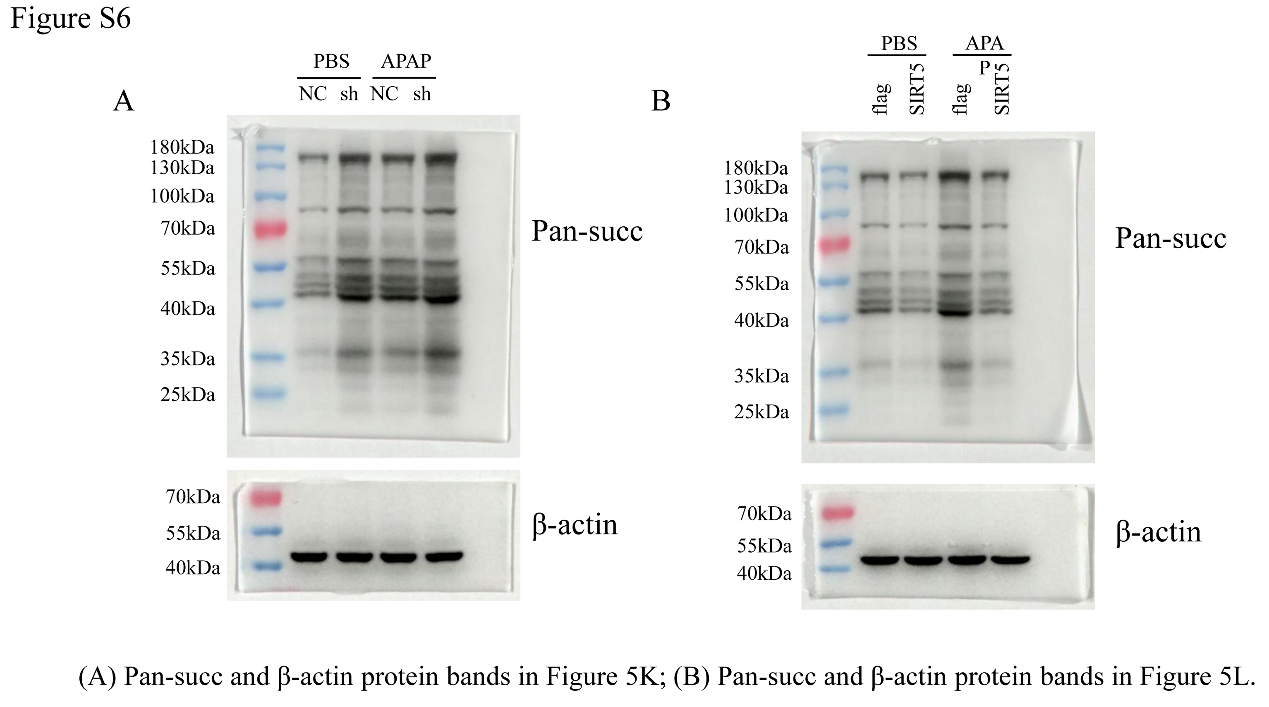

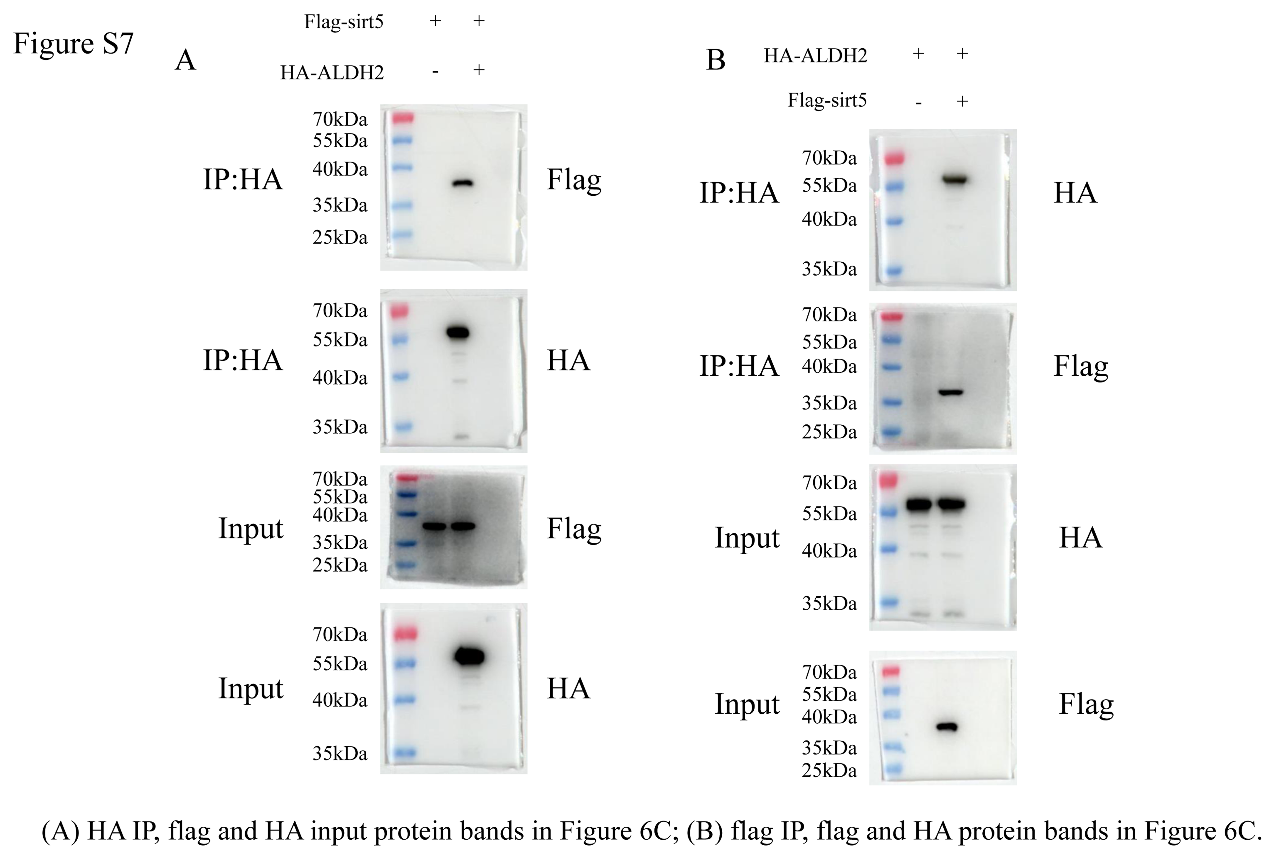


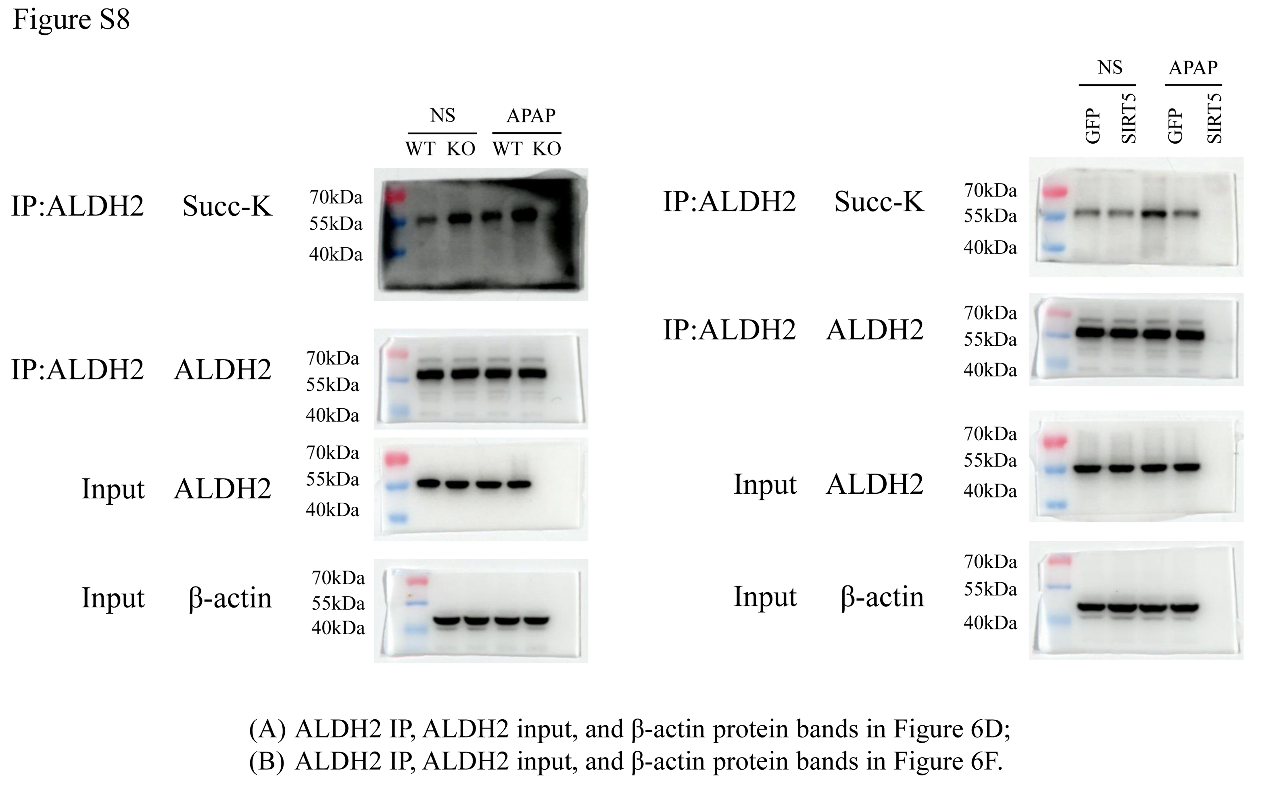

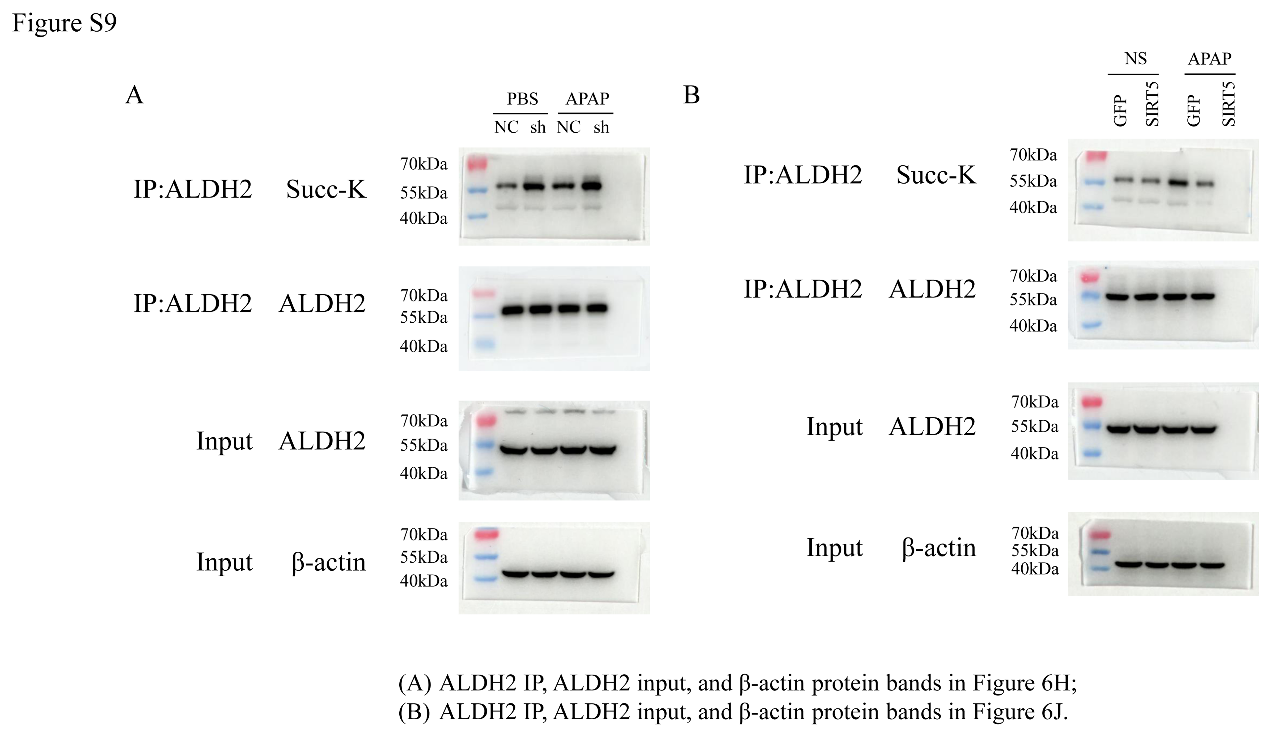


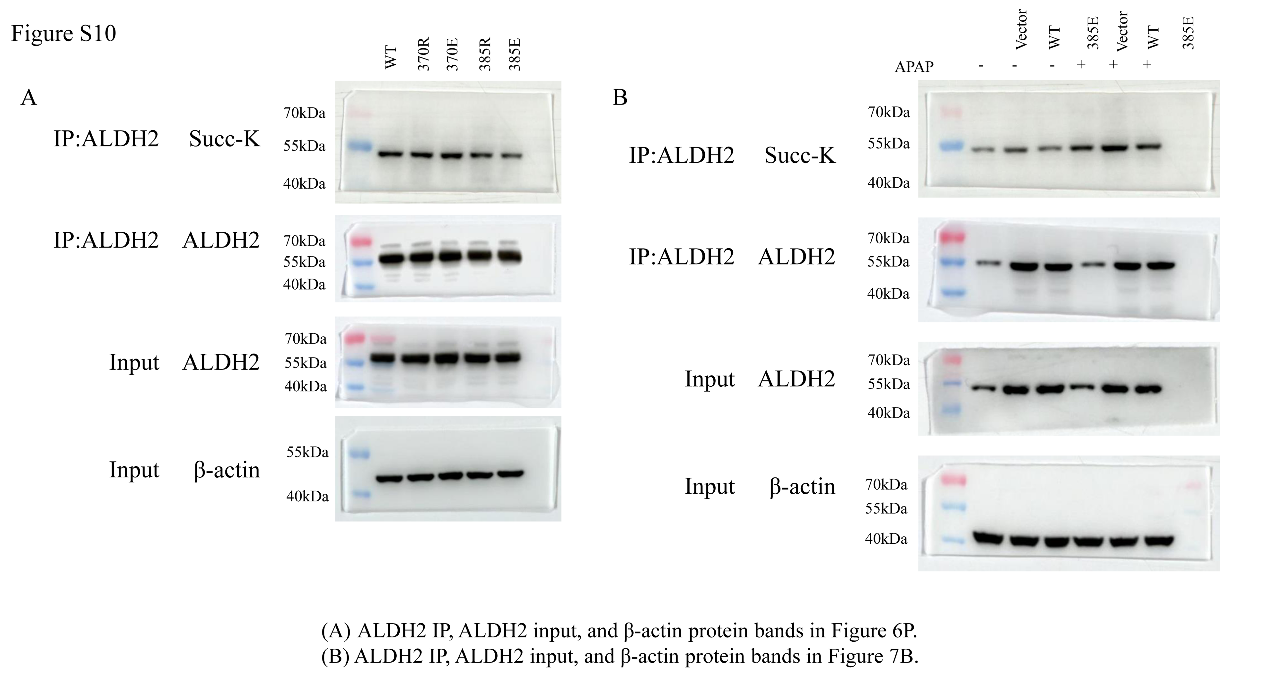

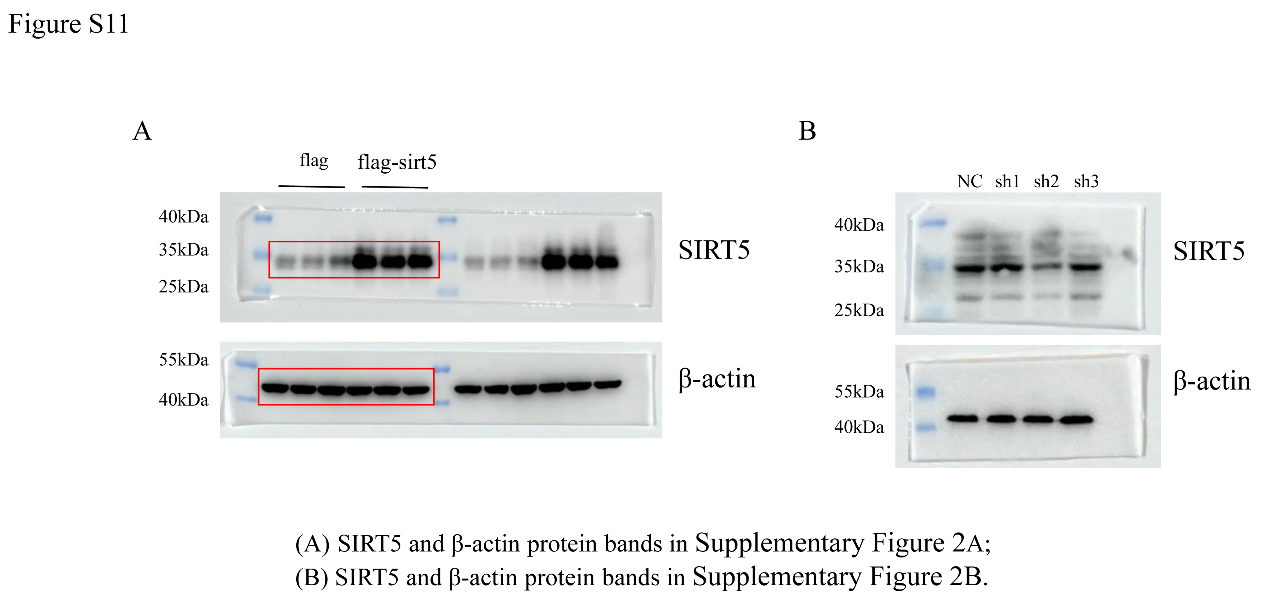

Supplement: Supplementary file 1 — Supporting Information [file ADVS-11-2402710-s001.docx]
